# Screen for Potential Candidate Alternatives of *Sargentodoxa cuneata* from Its Six Adulterants Based on Their Phenolic Compositions and Antioxidant Activities

**DOI:** 10.3390/ijms20215427

**Published:** 2019-10-31

**Authors:** Lingguang Yang, Peipei Yin, Xinxin Cao, Yujun Liu

**Affiliations:** 1 Jiangxi Provincial Key Laboratory of Natural Active Pharmaceutical Constituents, Yichun University, Yichun 336000, China; yanglingguangxdjqz@163.com (L.Y.); happy62889@126.com (P.Y.); 2 National Engineering Laboratory for Tree Breeding, College of Biological Sciences and Biotechnology, Beijing Forestry University, Qinghuadonglu No. 35, Haidian District, Beijing 100083, China; 15501080616@163.com

**Keywords:** *Sargentodoxa cuneata* (Oliv.) Rehd. et Wils, medicinal linana, adulterants, potential candidate alternatives, phenolics, antioxidant activity, UPLC-DAD-QTOF-MS/MS analysis, HPLC fingerprint

## Abstract

Daxueteng, the liana stem of *Sargentodoxa cuneata*, is a widely used Traditional Chinese Medicine facing the overflow of its commercial adulterants. A method for discriminating adulterants and screening potential candidate alternatives of *S. cuneata* was thus established. Total phenols and flavonoids of *S. cuneata* and its six adulterants and their abilities to scavenge DPPH^•^ and ABTS•+, to absorb peroxyl radicals (ORAC), and to inhibit AAPH-induced supercoiled plasmid DNA strand scission were comprehensively assessed. *Polygonum cuspidatum* and *Bauhinia championii*, two of the six adulterants of *S. cuneate*, shared considerably higher antioxidant activities as well as phenolic contents and, therefore, were considered as potential candidate alternatives. Phenolic compositions of the two potential candidate alternatives and *S. cuneata* itself were further determined by UPLC-QTOF-MS/MS. Totally 38 phenolics, including four hydroxybenzoic acids, two tyrosols, two caffeoylquinic acids, seven flavanol or its oligomers, two lignans, three hydroxycinnamic acids, six stilbenes, seven anthraquinones, and five flavanones were determined from three species. Furthermore, contents of different phenolic categories were semi-quantified and the major antioxidant contributors of *S. cuneata* and the two potential candidate alternatives were subsequently determined. It is concluded that tyrosols and caffeoylquinic acids were unique categories making great antioxidant contributions in *S. cuneata* and thus were considered as effective biomarkers in distinguishing its potential candidate alternatives.

## 1. Introduction

*Sargentodoxa cuneata*, a deciduous woody liana that belongs to Sargentodoxaceae (previously attributed to Lardizabalaceae), widely distributed over mountain forests from East Asia to central China [1] and sporadically occurred in Laos and Northern Vietnam [2]. According to the Pharmacopoeia of People’s Republic of China [3], the liana stem of *S. cuneata* has long been utilized as heat-clearing folk medicine named “Daxueteng” in China for its significant anti-inflammatory [4], anti-thrombotic, anti-tumor [5], anti-bacterial [6], anti-viral [7], and anti-allergic [8] effects. Furthermore, Li et al., [9] suggested that *S. cuneata* exhibited the highest total phenols and antioxidant capacities among 45 selected medicinal plants.

The World Health Organization (WHO) has estimated that over 80% of the earth’s inhabitants rely on the plant extracts and their active components of herbal medicines for their primary health care needs [10]. According to a report from Global Industry Analysts, the demand for herbal medicine increased with compound annual growth rate (CAGR) of 6–10% and this market is forecasted to reach $107 billion by the year 2017 (http://www.strategyr.com/MarketResearch). Data from the National Bureau of Statistics of China also indicated that the production of herbs used for Chinese Traditional Medicine reached 3.5 million tons in 2015 and over 3.7 million tons in 2016 (http://data.stats.gov.cn). With such a large production scale, pharmacovigilance is urgently required for those herbal medicinal products and drug safety should not be considered secondary to efficacy or be compromised by this increasing demand [10]. One of the major safety problems always faced by herbal drugs is the appearance of their commercial adulterants owing to the difficulty to identify herbs visually, especially for liana herbal medicine which occurred in markets as dry slices of their stems. Undeclared chemical and active ingredients of the adulterants could result in various degrees of side effects [11], and one of the most appalling examples is that the adulterants of herbal medicines have induced seriously anticholinergic poisoning in Hong Kong during 1989–2012 [11]. Specific to *S. cuneata*, the natural resources of *S. cuneata* have been declining dramatically due to long-term harvest, which particularly led to the overflow of its adulterants [12]. Therefore, it is essential to establish a rapid method for distinguishing *S. cuneata* from its adulterants and for screening its potential candidate alternatives from authentic herbs possessing similar medical effects.

In previous studies, many sound methods and tools were reported for the screening of adulterants for herbs or foods. Among which, liquid chromatography-diode array detector-quadrupole time-of-flight-mass spectrometer/mass spectrometer (LC-DAD-QTOF-MS/MS) [13,14,15] and DNA barcoding [16,17] were frequently applied. DNA barcoding is able to judge the relationship of various species at the genetic level. However, it requires elaborate sample preparation starting from fresh plants rather than processed herbal products like dry stems of *S. cuneate* occurring in the market. Comparing with DNA barcoding, LC-DAD-QTOF-MS/MS requires less sample condition and skill of operator, and thus contribute to the discovery of chemical constituents in crude herbal extracts and makes the overall comparison of chemical composition among authentic herbs and their adulterants possible.

Recently, studies have been focused on isolation and identification of bioactive components in *S. cuneata*, and several types of compounds including phenolics [18,19], lignans [19], triterpenes [20], anthraquinones [21], and phenylpropanoids [19] were characterized. Chang and Case [22] demonstrated that among 12 compounds isolated from the water-soluble constituents of *S. cuneata* stem, only two phenolic glycosides (cuneatasides A and B) possessed significant inhibitory activity against two gram-positive organisms, *Staphylococcus aureus* and *Micrococcus epidermidis*. Meanwhile, Zeng et al., [6] also suggested that hydroxytyrosol, a phenolic acid, possessed the highest antibacterial activity against *S. aureus* and *Acinetobacter baumannii* among 39 isolated compounds from the stem of *S. cuneata*. These researches indicated that phenolics in *S. cuneata* contribute not only to the well-known antioxidant ability but also to the antibacterial activity. Meanwhile, as inflammatory responses often include formation of tissue-damaging oxidation products, antioxidant ability could also be correlated to anti-inflammatory activity [23]. Therefore, phenolics could be selected as marker components for establishing methods for distinguishing adulterants and screening alternatives of *S. cuneata*.

In the present study, in order to screen adulterants and potential candidate alternatives of *S. cuneata* efficiently, total phenols and flavonoids and overall antioxidant activities of *S. cuneata* and its six adulterants were comprehensively assessed. Furthermore, an efficient HPLC fingerprint was developed for distinguishing *S. cuneata* from its adulterants, *Polygonum cuspidatum* and *Bauhinia championii* were determined as potential candidate alternatives for *S. cuneata*, and their antioxidant contributors were identified and semi-quantified with LC-DAD-QTOF-MS/MS. To the best of our knowledge, this is the first report of a method for screening adulterants and potential candidate alternatives of *S. cuneata*, and thus provide an efficient guideline for its industrial utilization.

## 2. Results and Discussion

### 2.1. Differences in Total Phenols and Flavonoids in Stems of S. cuneata and Its Six Adulterants

**Figure 1:**
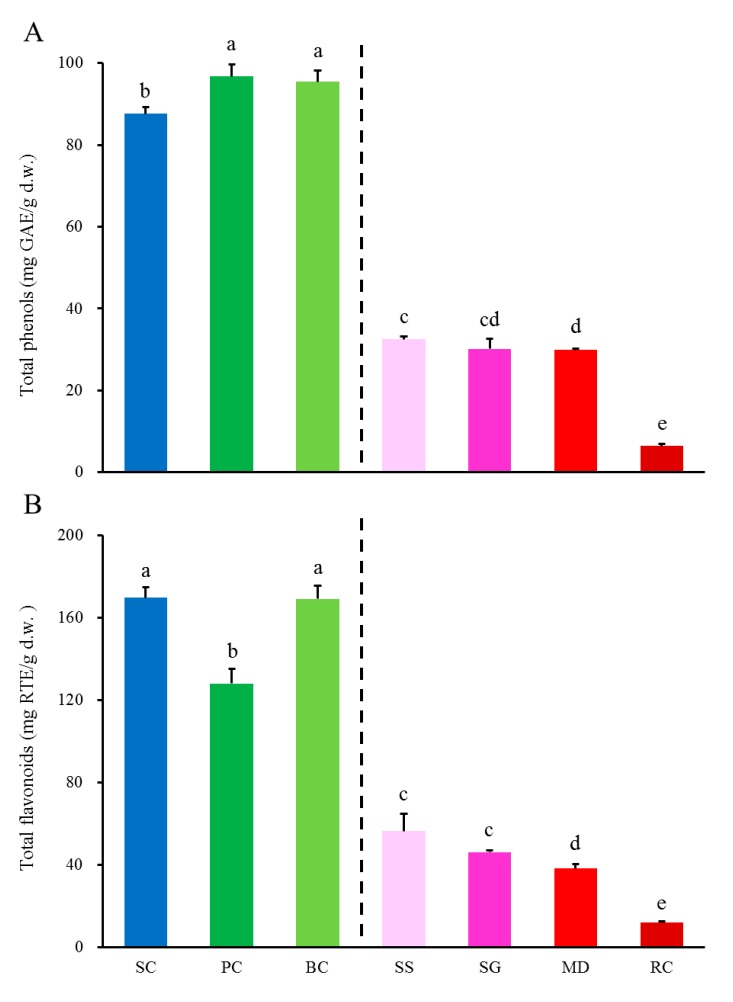
Total phenols (**A**) and total flavonoids (**B**) of *S. cuneata* and its six adulterants. SC, PC, BC, SS, SG, MD, and RC were abbreviations of *Sargentodoxa cuneata*, *Polygonum cuspidatum*, *Bauhinia championii*, *Schisandra sphenanthera*, *Schisandra grandiflora*, *Millettia dielsiana*, and *Rubia cordifolia* in respective. Vertical lines provide hints that data on the left side are far greater than those on the right side. Different letters mean significant difference (*p* > 0.05), and data are mean ± SD (*n* = 3).

The Folin-Ciocalteu method was applied in determining total phenols in stems of *S. cuneata* and its six adulterants. As shown in Figure 1A, different samples exhibited diverse levels of total phenols with an order as *Polygonum cuspidatum* (PC) and *Bauhinia championii* (BC) > *Sargentodoxa cuneate* (SC) >> *Schisandra sphenanthera* (SS), *Schisandra grandiflora* (SG) and *Millettia dielsiana* (MD) > *Rubia cordifolia* (RC), namely, total phenols of SC were significantly lower than those of PC and BC, but far higher (5.03-fold in average) than all other four samples (SS, SG, MD, and RC) as hinted by the vertical dashed line. It should be noted that total phenols of SC determined in the present study (87.59 ± 1.56) were much higher compared with those determined by Li et al., [9] (52.35 ± 0.25), which was much likely due to a higher extraction efficiency caused by our ultrasonic method.

Figure 1B shows total flavonoids analyzed by aluminium chloride colorimetric assay with a similar order to that of total phenols as SC, BC > PC >> SS and SG > MD > RC. In other words, total flavonoids of SC, BC and PC were still far higher (5.44-fold in average) than those of SS, SG, MD and RC as hinted also by the vertical line. 

Interestingly, in the group at the left side of the two vertical lines of Figure 1, PC exhibited the lowest total flavonoids (Figure 1B) but the highest total phenols (Figure 1A), and SC exhibited the highest total flavonoids (Figure 1B) but the lowest total phenols (Figure 1A), indicating that there existed divergent phenolic compositions in the three species (SC, PC, and BC) and total flavonoids were the major contributor to the total phenols in SC.

### 2.2. Differences in Antioxidant Activities in Stems of S. cuneata and Its Six Adulterants

#### 2.2.1. DPPH^•^ and ABTS•+ Scavenging Activities and ORAC

**Figure 2:**
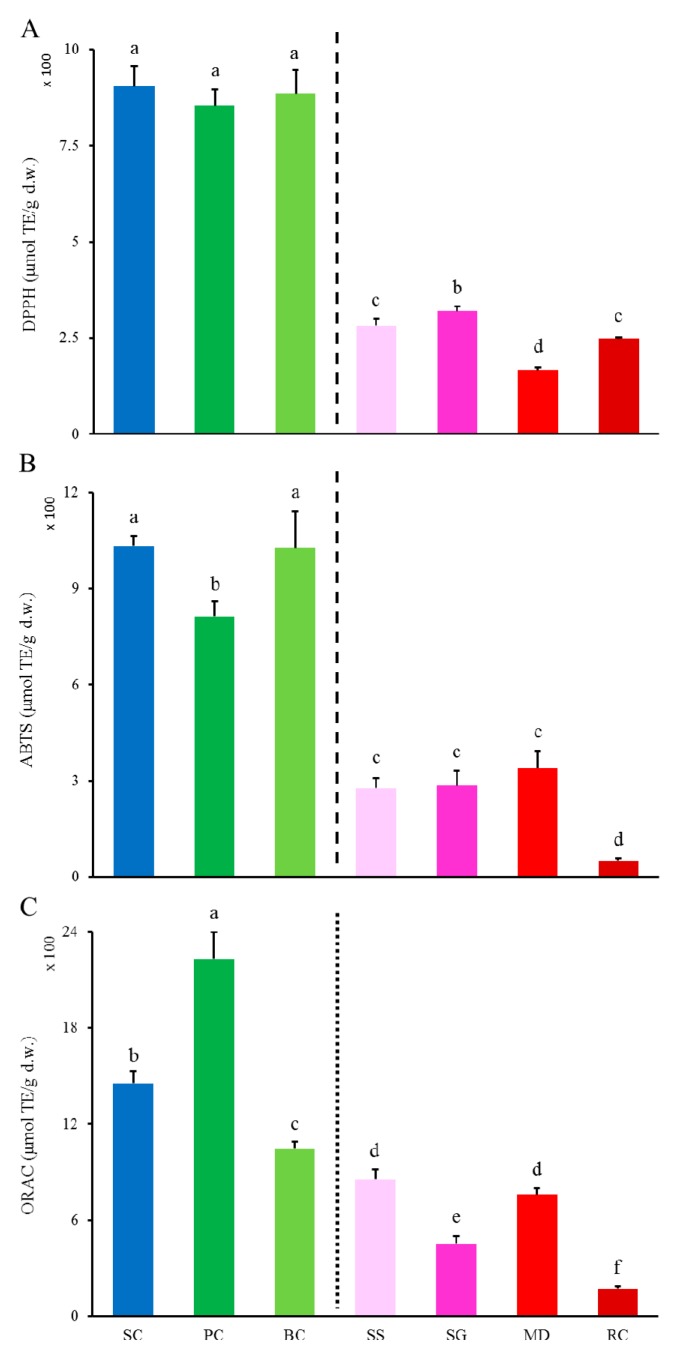
Antioxidant activities of S. cuneata and its six adulterants determined with DPPH (**A**), ABTS (**B**), and ORAC (**C**) assays. SC, PC, BC, SS, SG, MD, and RC were abbreviations of *Sargentodoxa cuneata*, *Polygonum cuspidatum*, *Bauhinia championii*, *Schisandra sphenanthera*, *Schisandra grandiflora*, *Millettia dielsiana*, and *Rubia cordifolia* in respective. Vertical dashed (**A**,**B**)/dotted (**C**) lines provide hints that data at the left side are far greater/greater than those on the right side. Different letters mean significant difference (*p* > 0.05). Data are mean ± SD (*n* = 3).

Radical scavenging abilities of *S. cuneata* and its six adulterants were determined with DPPH and ABTS assays. As shown in Figure 2A,B, DPPH^•^ scavenging abilities ranked as SC, BC and PC >> SG > SS and RC > MD and SC, and ABTS•+ scavenging abilities ranked as SC and BC > PC >> MD, SG, and SS > RC. Most notably, the two-levels pattern found in both total phenols and flavonoids (Figure 1) also occurred in DPPH^•^ and ABTS•+ scavenging abilities as hinted also by the dashed lines, with the left side group (i.e., SC, BC, and PC) were far higher (4.62-fold and 5.35-fold in averages in respective) than those of the right side group (i.e., SG, SS, RC and MD), indicating that total flavonoids were key constituents of total phenols, and total phenols were main contributors to radical scavenging abilities of *S. cuneata* and its six adulterants, especially those two (i.e., PC and BC) in the left side group. ABTS•+ as shown in Figure 2C, similar order to those of DPPH^•^ and ABTS•+ scavenging abilities was not found when measuring ORAC which ranked as PC > SC > BC > SS and MD > SG > RC. Nevertheless, both PC, SC, and BC still showed higher values (3.74-fold on average) than those of the other four as arbitrarily divided by the fine vertical dotted line. This distinction could be interpreted by the mechanistic divergence between ORAC and DPPH or ABTS assays. To be more specific, radicals are mainly quenched via two chemical principles, namely, transfer of either a hydrogen atom or a single electron to convert radicals into a stable form [24]. DPPH and ABTS assays are based on the transfer of single electron and measures the electron-donating capacity of antioxidants to reduce DPPH^•^ and ABTS•+ radicals. In the case of the ORAC assay, antioxidants slow the loss of fluorescence by quenching the peroxyl radicals produced by AAPH via transfer of hydrogen atom or radical addition. The difference in principles between these two types of mechanisms might be responsible for the variation between the rank order of *S. cuneata* and its six adulterants in the three assays for antioxidant abilities.

#### 2.2.2. Differences in Inhibition against Radicals-Induced Supercoiled Plasmid DNA Strand Scission

**Figure 3:**
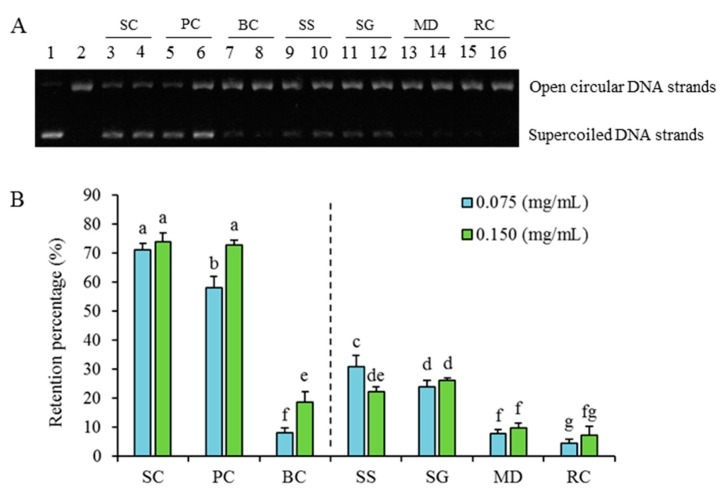
Agarose gel electrophoretograms of AAPH-induced supercoiled plasmid DNA strand scission (**A**) and their correspondingly calculated retention percentages (**B**). Lanes treated with PBS (lane 1), AAPH (lane 2), and AAPH + phenolic extracts of *S. cuneata* and its six adulterants (lanes 3-16) were presented. From lanes 3-16, lanes labeled with odd numbers were treated with extracts at a concentration of 0.075 mg/mL, and those labeled with even numbers were treated with those at a concentration of 0.150 mg/mL. SC, PC, BC, SS, SG, MD, and RC were abbreviations of *Sargentodoxa cuneata*, *Polygonum cuspidatum*, *Bauhinia championii*, *Schisandra sphenanthera*, *Schisandra grandiflora*, *Millettia dielsiana*, and *Rubia cordifolia*, respectively. Different letters in (**B**) mean significant difference (*p* > 0.05).

It is well known that overproduction of reactive oxygen species (ROS) [arising either from mitochondrial electron transport chain or excessive stimulation of NAD(P)H] leads to oxidative stress that results in deleterious damage to cell structures, including lipids and membranes, proteins, and DNA [25]. Under ROS stresses, the supercoiled plasmid DNA converts to open circular DNA followed by linear form via single or double-strand breaks, and this conversion could be used as an indicator of DNA damage [26]. In the present study, supercoiled plasmid DNA under AAPH-induced oxidative stress was treated with extracts from *S. cuneata* and its six adulterants. As shown in Figure 3A, DNA bands in supercoiled and open circular form were well separated, and different samples exhibited diverse inhibition abilities against supercoiled plasmid DNA strand scission. The calculated retention percentages of AAPH-induced supercoiled plasmid DNA strand scission were presented in Figure 3B. Results show that SC both at 0.075 and 0.150 mg/mL and PC at 0.150 mg/mL exhibited the highest protective effects. The inhibition ability of PC at 0.075 mg/mL was significantly lower than those of the above three but was still significantly far higher than all other treatments at two concentrations of the other five species. In contrast to the results of phenolic and flavonoids contents (Figure 1) and the three antioxidant abilities (Figure 2), the protective effect of BC was much lower comparing with the other two species (SC and PC) in the former higher-level group and was even lower than those of two in the lower-level group (SS and SG) as divided by the vertical lines.

As phenols were demonstrated as key bioactive compounds and antioxidant ability could be correlated to anti-inflammatory activity in SC, PC and BC possessed the highest potential of being candidate alternatives of SC because of their similar higher total phenols, total flavonoids, and antioxidant abilities (Figure 1 and Figure 3). Among which, PC might be better than BC based further on the results of their ORAC values (Figure 2C) and inhibition abilities on radicals-induced supercoiled plasmid DNA strand scission.

### 2.3. Differences in HPLC Fingerprints of S. cuneata and Its Six Adulterants

**Figure 4:**
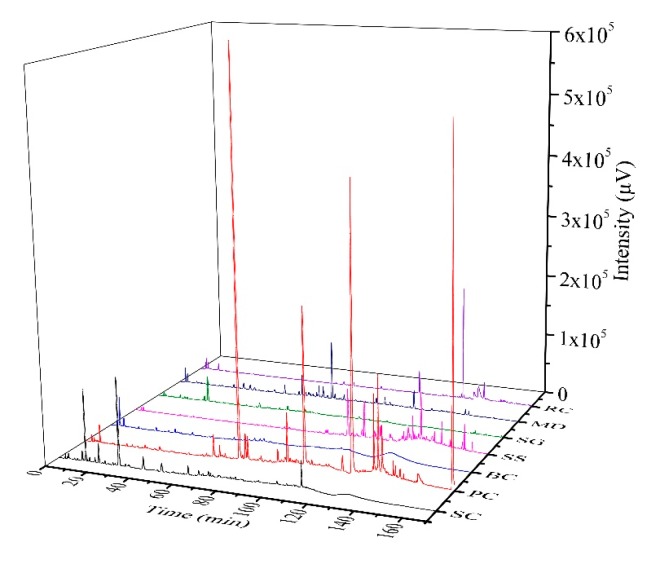
HPLC fingerprints of S. cuneata and its six adulterants. SC, PC, BC, SS, SG, MD, and RC were abbreviations of Sargentodoxa cuneata, Polygonum cuspidatum, Bauhinia championii, Schisandra sphenanthera, Schisandra grandiflora, Millettia dielsiana, and Rubia cordifolia, respectively.

To further determine the detailed phenolic composition of *S. cuneata* and its adulterants, an HPLC fingerprint that suits for all samples was established. As shown in Figure 4, peaks were well separated in each sample, and all of them exhibited a diverse HPLC profile. Comparing with the profile of SC with those of its six adulterants by Similarity Evaluation System for Chromatographic Fingerprint of Traditional Chinese Medicine software (Version 2004 A), none of the adulterants showed significant correlations with SC. Therefore, it could be concluded that the phenolic composition of all those adulterants, even the two possessing highest potential of being candidate alternatives (i.e., PC and BC) screened based on the above data, varied a lot from that of SC. Consequently, more detailed information on specific phenolic compounds in SC and its two potential candidate alternatives (PC and BC) needs to be further identified by the MS/MS technology, and antioxidant contributions of individual phenolics should be subsequently analyzed.

### 2.4. Characterization of Phenolic Compounds by UPLC-DAD-QTOF-MS/MS in S. cuneata and Its Two Potential Candidate Alternatives

**Figure 5:**
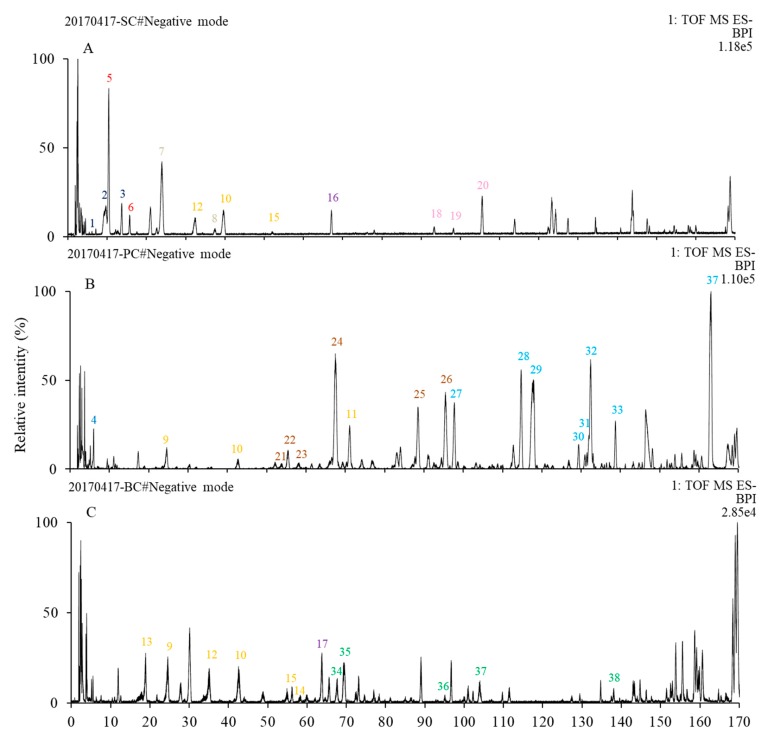
UPLC-QTOF-MS spectra of SC (**A**), PC (**B**) and BC (**C**). Numbers in (**A**–**C**) correspond to those peak numbers in Table 1. SC, PC, and BC were abbreviations of *S. cuneata*, *P. cuspidatum*, and *B. championii*, respectively.

Although SC and its two potential candidate alternatives (i.e., PC and BC) exhibited similar DPPH, ABTS, and ORAC values as well as total phenols and flavonoids, varied phenolic profiles among them could still lead to diverse medical effects. Therefore, UPLC-DAD-QTOF-MS/MS was applied to further characterize their phenolic compositions, and to establish screening parameters for identifying their commercialized products. Both positive and negative ion modes were used aiming to obtain appropriate ionization. The results indicate that the negative ion mode possessed cleaner parent and fragment ion signals, better resolution, and lower background noise compared with the corresponding positive ion mode, which is likely due to the acidic nature of the detected compounds. Therefore, analyzes were conducted with the data obtained from the negative ion mode. The MS spectra of SC, PC, and BC were presented in Figure 5A–C, respective. It could be observed that the spectra obtained from different samples were quite complex, and 14, 17 and 12 phenolic compounds were tentatively identified from SC, PC, and BC, respectively.

Table 1 shows a list of totally 38 compounds tentatively identified through UPLC–DAD–QTOF-MS/MS along with their peak numbers, maximal UV absorptions, precursor ions, error values, predictive formula, MS/MS ions, compound names, compound group classified based on their structure, the samples where they were collected, and the references or MS database supporting the identification. Totally, among these 38 phenolics, four were identified as hydroxybenzoic acids, two are tyrosols, two are caffeoylquinic acids, seven are flavanol or its oligomers, two are lignans, three are hydroxycinnamic acid, six are stilbenes, seven are anthraquinones, and five are flavanones.

*Four hydroxybenzoic acids*: Compounds 1–4 were identified as hydroxybenzoic acid derivatives as they all exhibited a characteristic fragment ion with the loss of a carboxyl group (CO_2_) [27]. Compound 1 was assigned as protocatechuic acid with a [M-H]^−^ at *m*/*z* 153 as it showed a fragment at *m*/*z* 109 with a loss of a CO_2_ (44 Da) [28]. Moreover, it exhibited similar maximal UV absorption with that reported by Liu et al., [29]. Compound 2 with a [M-H]^−^ at *m*/*z* 167 was identified as vanillic acid as it showed fragment ions at *m*/*z* 152 due to the loss of a CH_3_ (15 Da), and at 123 with another loss of a CO_2_ (44 Da) [27]. Compound 3 with a [M-H]^−^ at *m*/*z* 197 was identified as syringic acid because it exhibited product ions at *m*/*z* 182 due to the loss of a CH_3_ (15 Da), at *m*/*z* 166 with the loss of another CH_3_, and at *m*/*z* 153 with the loss of a CO_2_ (44 Da) [30]. Compound 4 was identified as gallic acid with a [M-H]^−^ at *m*/*z* 169 as it showed a characteristic fragment at *m*/*z* 125 with the loss of a CO_2_ (44 Da) [31].

*Two tyrosols*: Compounds 5 and 6 both exhibited similar maximum UV absorbance at 228–230 and 276–280 nm. Therefore, it could be deduced that they shared similar structures. Compound 5 with a [M-H]^−^ at *m*/*z* 315 was assigned as hydroxytyrosol-1-*O*-glucoside because it showed fragment ions at *m*/*z* 153, which was the fragment ion of hydroxytyrosol [32]. Compound 6 with a [M-H]^−^ at *m*/*z* 299 was assigned as salidroside as its fragmentation patterns corresponded with those reported by Han et al., [33].

Two caffeoylquinic acids: Compounds 7 and 8 both showed a [M-H]^−^ at *m*/*z* 353 and maximum UV absorbances at around 245 and 320 nm, thus they were identified as caffeoylquinic acid isomers. It is reported that compounds with similar chemical structures, like isomers or aglycone with different glycosides, always eluted in the same order in C_18_ column [34]. Based on their MS/MS fragments and elution order in the C_18_ column, compound 7 was identified as 3-*O*-caffeoylquinic acid, and compound 8 was identified as 5-*O*-caffeoylquinic acid [35].

Seven flavanols or its oligomers: Compounds 9-15 all showed two characteristic maximum UV absorbances at about 240 and 280 nm, indicating that they might be flavanols or its oligomers. Compounds 9 and 10 shared the same parent ion at *m*/*z* 289 and fragment ions at *m*/*z* 137 and 109. Due to their elution order in the C_18_ column, Compound 9 was identified as catechin and compound 10 was assigned as epicatechin [36,37]. Compound 11 with a [M-H]^−^ at *m*/*z* 441 was assigned as catechin-gallate due to its fragmentation at *m*/*z* 289 (catechin moiety) and *m*/*z* 169 (gallic acid moiety) [38]. Compounds 12–14 exhibited the same parent ion at *m*/*z* 577 and characteristic product ions at *m*/*z* 289 and 287. As previously reported, proanthocyanidins yielded two different types of ions in the collision-induced MS analysis depending on whether they possessed the extension (287 Da) or terminal units (289 Da) of the corresponding proanthocyanidin molecule [4,39]. Therefore, compounds 12–14 were identified as B-type proanthocyanidin dimers 1–3. With the same fragmentation patterns, compound 15 with a [M-H]^−^ at *m*/*z* 865 was identified as B-type proanthocyanidin trimer.

Two lignans: Compound 16, with a [M-H]^−^ at *m*/*z* 579 and a characteristic fragment at *m*/*z* 417 (syringaresinol moiety), was identified as syringaresinol-4-*O*-glucoside [22,40]. Compound 17 with a [M-H]^−^ at *m*/*z* 581 was assigned as lyoniresinol glucoside as it fragmented at *m*/*z* 419 (lyoniresinol moiety).

Three hydroxycinnamic acids: Compounds 18 with a [M-H]^−^ at *m*/*z* 359 was identified as rosmarinate as it fragmented at *m*/*z* 197 (Danshensu moiety) [29]. Compound 19 with a [M-H]^−^ at *m*/*z* 717 fragmented at *m*/*z* 519 with a loss of a Danshensu moiety (198 Da) and at *m*/*z* 321 with another loss of a Danshensu moiety (198 Da), thus was identified as salvianolic acid B. Following the same fragmentation patterns, compound 20 with a [M-H]^−^ at *m*/*z* 493 was identified as salvianolic acid A.

Six stilbenes: Compound 26 with a precursor ion at *m*/*z* 227 was identified as resveratrol because it showed a product ion at *m*/*z* 185 representing a loss of a ketene molecule [41]. Meanwhile, compounds 21–25 all shared a resveratrol moiety (227 Da), indicating that they were resveratrol derivatives. Compounds 21 and 23 with a [M-H]^−^ at *m*/*z* 469 were assigned as resveratrol-sulfoglucoside isomers, and compounds 22 and 24 with a [M-H]^−^ at *m*/*z* 469 were identified as resveratrol-4′-glucoside (resveratroloside) and resveratrol-3-*O*-glucoside (piceid), respectively [38]. Compound 25 with a [M-H]^−^ ion at *m*/*z* 541 was identified as resveratrol-galloylglucoside as it showed a fragment ion at *m*/*z* 169 (gallic acid moiety) [38].

Seven anthraquinones: Compound 32, with a precursor ion at *m*/*z* 299 and product ions at *m*/*z* 255 and 277 were identified as emodic acid, which corresponded with the data reported by Yao et al., [42]. Compound 33 with a [M-H]^−^ at *m*/*z* 269 was identified as emodin as it showed a fragment ion at *m*/*z* 225 due to the loss of a CO_2_ (44 Da) [38]. With a characteristic ion at *m*/*z* 269, compounds 27-29 were assigned as emodin derivatives. Comparing their fragmentation patterns with those of literature compounds 27 and 28 with a [M-H]^−^ at *m*/*z* 431 were identified as emodin-8-*O*-glucoside and emodin-1-*O*-glucoside in respective, and compound 29 with a [M-H]^−^ at *m*/*z* 517 was assigned as emodin-8-*O*-(6′-*O*-malonyl)-glucoside [38]. Compound 30 with a [M-H]^−^ at *m*/*z* 283 was identified as physcion since it exhibited a fragment at *m*/*z* 268 due to a loss of a CH_3_ (15 Da). Compound 31, with a [M-H]^−^ at *m*/*z* 285, fragmented at *m*/*z* 267, 257 and 255, which corresponded with those recorded in the METLIN database (https://metlin.scripps.edu), was identified as citreorosein.

Five flavanones: Compounds 37 and 38, with the same parent ion at *m*/*z* 255 and daughter ion at *m*/*z* 119, were assigned as liquiritigenin isomers. Compounds 34–36 exhibited the same precursor ion at *m*/*z* 417 and product ion at *m*/*z* 255, thus were assigned as liquiritin isomers. Based on their elution order in the C_18_ column reported by Wang et al., [37], compounds 34–38 were identified as liquiritin, neoliquiritin, neoisoliquiritin, liquiritigenin and isoliquiritigenin in respective.

To validate the accuracy of our tentatively identification, 16 of the 38 phenolics (i.e., compounds 4, 6, 8, 10, 12, 14, 18, 19, 20, 26, 27, 28, 33, 35, 37, 38) were verified by comparing with their standards (Table 1 and Figure S1). 

### 2.5. Antioxidant Contribution of Key Individual Phenolics in S. cuneata and Its Two Potential Candidate Alternatives

In order to unearth potential contributors to overall antioxidant activity in SC, PC and BC, and to screen markers for discriminating *S. cuneata* from its two potential candidate alternatives, antioxidant activities of nine representative phenolic compounds (or their aglycones) from the eight detected phenolic categories (Table 1) described above were evaluated with DPPH, ABTS, and ORAC assays. Furthermore, content for every single phenolic category measured by semi-quantification and its corresponding antioxidant contribution was subsequently calculated. For instance, gallic acid was selected as the representative phenolic compounds from hydroxybenzoic acids category, and its DPPH, ABTS, and ORAC values were determined as 3.98 ± 0.38, 6.08 ± 0.36, and 2.23 ± 0.03 (μmol Trolox equivalent/μmol) in respective (Table 2). Meanwhile, all hydroxybenzoic acids in SC were semi-quantified by comparing their peak areas with that of gallic acid, and the sum of all these hydroxybenzoic acids was defined as their semi-content. After that, antioxidant contribution of hydroxybenzoic acids in SC was obtained by multiplying their semi-content to DPPH, ABTS or ORAC values of gallic acid.

As shown in Table 2, the nine representative phenolics exhibited diverse DPPH^•^ scavenging abilities with a rank order as gallic acid > epicatechin > chlorogenic and rosmarinic acid > resveratrol > proanthocyanidin B2 > tyrosol > liquiritigenin > emodin. Gallic acid showed a significantly higher DPPH value comparing with other phenolics, while emodin exhibited no DPPH^•^ scavenging ability comparing with others under the same concentrations. As for ABTS values, the nine phenolics exhibited a similar rank order to that of DPPH, and gallic acid still exhibited the highest ABTS•+ scavenging ability, indicating that hydroxybenzoic acids might possess higher electron-donating capacities comparing with other phenolic categories. In contrast, gallic acid only ranked the second to last among the nine phenolics in ORAC, and proanthocyanidin B2 possessed the highest ORAC value, followed by chlorogenic acid and rosmarinic acid. Tabart et al., [44] reported that gallic acid possesses a relatively similar activity in ABTS and ORAC assays while other phenolics including flavonols, flavan-3-ols, and flavanons exhibited a much higher ORAC value than ABTS value, supporting results of the present study.

SC possessed phenolics in six categories while PC and BC only have those in four and three categories, respectively. Caffeoylquinic acids, tyrosols, and hydroxycinnamic acids occurred only in SC while stilbenes and anthraquinones only existed in PC and flavanones were a unique category for BC (see dark red words in Table 2). In SC, hydroxybenzoic and caffeoylquinic acids and tyrosols made important contributions to overall antioxidant capacities as each of them made the largest contribution in one assay. As for PC, hydroxybenzoic acids, flavanols, and stilbenes played important roles in contributions to DPPH and ORAC, but anthraquinones ranked the first in ORAC assay. In BC, flavanols made the most contributions to the overall DPPH^•^ and ABTS•+ scavenging abilities, and both flavanols and its oligomers contributed to the most of ORAC. Sum of the contributions made by all phenolic categories in an individual species ranged from 21.32% to 87.80% in DPPH and ABTS assays, but from 197.22% to 430.42% in ORAC assay. Those 1.97- and even 4.30-fold higher contributions comparing with overall ORAC values might partially result from the deviation caused by the semi-quantification, but more likely owing to an existing antagonistic effect. The reason for saying this is because the excessive levels of ORAC contributions occurred in all three species. Ciesla et al., [45] explored the antagonistic antioxidant interactions of common monoterpenes with DPPH assay, which verified that the antagonism effect existed in many combinations. Little information was gained concerning antagonism effect of phenolics for ORAC assays, thus more detailed experiments need to be conducted to testify the conjecture.

Considering tyrosols and caffeoylquinic and hydroxycinnamic acids occurred only in SC with the former two exhibiting relative higher antioxidant activity contributions in all the three assays, tyrosols and caffeoylquinic acids should be effective biomarkers for SC in distinguishing its alternatives.

## 3. Materials and Methods

### 3.1. Chemicals, Reagents and Plant Materials

All authentic standards were purchased from the National Institutes for Food and Drug Control (Beijing, China). Folin-Ciocalteu reagents, 6-hydroxy-2,5,7,8-tetramethylchroman-2-carboxylic acid (Trolox), 2,2′-diphenyl-1-picrylhydrazyl (DPPH), 2,2′-azobis-(3-ethylbenzothiazoline-6-sulfonic acid) (ABTS), 2,2′-azobis (2-methylpropionamidine) dihydrochloride (AAPH), and fluorescein were bought from Sigma Chemical (St. Louis, MO, USA). Supercoiled plasmid DNA (pBR 322 from E. coli) was purchased from Thermo Fisher Scientific Inc. (Waltham, MA, USA). Tris-acetate-EDTA (TAE) buffer and GelRedTM nucleic acid stain were obtained from Biotium Inc. (California, USA). HPLC-grade acetonitrile and formic acid were bought from Fisher Scientific (Pittsburgh, PA, USA). Other reagents (analytical grade) were purchased from Sinopharm Chemical Reagent Co. Ltd. (Beijing, China).

**Figure 6:**
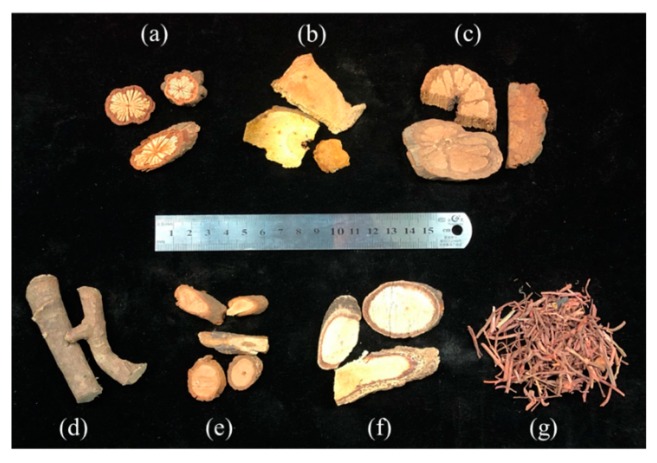
Herb configurations: *S. cuneata* (**a**), *P. cuspidatum* (**b**), *B. championii* (**c**), *S. sphenanthera* (**d**), *S. grandiflora* (**e**), *M. dielsiana* (**f**), and *R. cordifolia* (**g**).

Dry stem slices of *S. cuneata* (Oliv.) Rehd. et Wils (namely, the authentic herbal medicine Daxueteng) were purchased from National Institutes for Food and Drug Control (Beijing, China). Seven herbs all being claimed as ‘Daxueteng’ were collected from different drugstores and tradesmen in Anguo herbal medicine market (Anguo, Hebei province, China), the biggest herbal medicine market in China. Experts from Capital Medical University identified these collected samples morphologically as seven species, i.e., *S. cuneata* (Oliv.) Rehd. et Wils., *P. cuspidatum* Sieb.et Zucc., *B. championii* (Benth.) Benth., *S. sphenanthera* Rehd. et Wils., *S. grandiflora* (Wall.) Hook. f. et Thoms, *M. dielsiana* Harms, and *R. cordifolia* L. (Table 3, Figure 6). After gentle cleaning, all collected samples were ground and passed a 250 × 250 μm^2^ sieve, then the powder was stored at −20 °C for subsequent extraction.

### 3.2. Extraction of Phenolics from Stems of S. cuneata and Its Six Adulterants

To prepare phenolic extracts, an ultrasonic-assisted extraction procedure was performed. To be specific, 15 mL of 80% methanol was added to 1.000 g dry powder of *S. cuneata* or each of its six adulterants, then the mixture was sonicated (KQ-300DE type, Kunshan Ultrasonic Instrument Co., Ltd., Kunshan, China) in a 300 W and 40 °C water bath for 30 min with occasional stirring. The sonicated mixture was filtrated by a 0.22 μm filter to obtain the supernatant. The extraction process was repeated twice more with the residue, and three supernatants were poured together and diluted to 45 mL with 80% methanol for further experiments.

### 3.3. Measurements of Phenolics and Assessments of Antioxidant Capacities

#### 3.3.1. Total Phenols and Total Flavonoids

Total phenols were determined according to the Folin–Ciocalteu method [46], and total flavonoids were estimated by the aluminium chloride colorimetric assay [47], which were modified and detailed described in detail in one of our previous report [48]. Results were expressed as mg gallic acid equivalent (GAE)/g d.w. and mg rutin equivalent (RTE)/g d.w., respectively.

#### 3.3.2. DPPH• and ABTS•+ Scavenging Activities and ORAC

DPPH• scavenging activity was determined as described by Brand-Williams et al., [49] and ABTS•+ scavenging capacity was evaluated by using the method reported by Re et al., [50], ORAC (oxygen radical absorbance capacity) assay was conducted according to a report by Sun et al., [51], and these three methods were modified and described in detail in one of our previous report [46]. Results were calculated by comparing the net AUC of each sample with that of the standard. All results were expressed as μmol Trolox equivalent (TE)/g d.w.

#### 3.3.3. Inhibition of Radicals-Induced Supercoiled Plasmid DNA Strand Scission

Inhibition activity against peroxyl radicals-induced supercoiled DNA strand scission was evaluated according to reports by Chandrasekara and Shahidi [52] and Liyana-Pathirana and Shahidi [53] with some modifications. Supercoiled plasmid DNA (pBR 322 from *E. coli* RRI, 50 μg/mL), Trolox (5 and 10 μM), phenolic extracts (0.075 and 0.15 mg/mL), and AAPH (9 mM) were all dissolved in a 75-μM phosphate buffer solution (PBS, pH 7.4). In an Eppendorf tube (200 μL), PBS, supercoiled plasmid DNA, and phenolic extract or Trolox, 4 μL for each, and 8 μL AAPH were added in order to determine the inhibitory activity against peroxyl radicals-induced scission. A blank control with DNA alone and a negative control being devoid of extracts or Trolox were prepared with each set of phenolic extracts tested. The mixture was then incubated at 37 °C for 1 h in darkness [54]. The loading dye (4 μL), consisting of 0.25% bromophenol blue, 0.25% xylene cyanol and 50% glycerol in distilled water, was added to the reaction mixture at the end of the incubation.

The samples were then electrophoresed using GelRed™ (100 μL/L) as a gel stain and agarose gel (0.7%, *w*/*v*) prepared in a Tris-acetic acid-EDTA (TAE) buffer (40 mM Tris acetate, 1 mM EDTA, pH 8.5). Submarine gel electrophoresis was run in the TAE buffer for 1.5 h at room temperature and 60 V with a PowerPac™ Basic Power Supply (Bio-Rad Laboratories, Inc., California, USA). The bands were visualized under the transillumination of UV light using Azure Biosystems C600 (Azure Biosystems Inc, CA, USA). Images were analyzed using Azurespot software (Azure Biosystems Inc, CA, USA) to quantify DNA scission. Protective effects of phenolic extracts were calculated using retention percentage of the normalized supercoiled DNA as given below: DNA retention % = intensity of supercoiled DNA with AAPH and extract/intensity of supercoiled DNA in blank × 100.

### 3.4. Characterization and Quantification of Phenolic Compositions by UPLC-DAD-QTOF-MS/MS and HPLC-UV

The UPLC-DAD-QTOF-MS/MS system was comprised of an Acquity Ultra-Performance Liquid Chromatography (UPLC) system (Waters, Milford, MA, USA), a DAD detector and a QTOF-MS mass spectrometer (Xevo G2-XS, Waters). The chromatographic separation was performed on a reversed phase column (Diamonsil C_18_ 5 μm 250 × 4.6 mm i.d., Dikma, China) with column temperature set at 30 °C. The mobile phase consisted of water with 0.4% formic acid (v:v) (A) and acetonitrile (B) under the following gradient program: 0–10 min, 5–7% B, 10–46 min, 7–10% B, 46–70 min, 10–16% B, 70–81 min, 16–17% B, 81–97 min, 17–21% B, 97–104 min, 21–23% B, 104–110 min, 23–25% B, 110–120 min, 25% B, 120–200 min, 25–80% B. The flow rate was set at 1 mL/min with an injection volume of 10 μL.

Mass spectra were recorded in the range of *m*/*z* 50–2000. MS experiments were performed both in positive and negative ionization modes under the following conditions: nitrogen drying gas flow, 10.0 L/min, nebulizer pressure, 45 psi, gas drying temperature, 370 °C, capillary and fragmentor voltage, 2.5 kV, MS/MS collision energies, 20 V. Peak identification was performed by comparing the UV and mass spectra and fragmentation ions to those reported by literatures and METLIN database (https://metlin.scripps.edu).

HPLC analyses were performed using a Shimadzu HPLC system (Shimadzu, Japan) equipped with two LC-10AT VP pumps, an SPDM20A UV-detector, and a SIL-20AC TH autosampler controlled by an analytical software (LC Solution-Release 1.23SP1). The reversed phase column, column temperature, solvent system, gradient program, as well as the flow rate were the same as those in the UPLC analysis described above. The flow rate was set at 1 mL/min with an injection volume of 10 μL. The detection wavelength was set at 280 nm to monitor more phenols simultaneously. At the end of each running, the column was flushed with 95% acetonitrile for 10 min to remove strongly retained constituents, and then equilibrated for 10 min under initial conditions.

Contents of gallic acid, tyrosol, chlorogenic acid, epicatechin, proanthocyanidin B2, rosmarinic acid, resveratrol, emodin, and liquiritigenin in each sample were quantified by comparing their peak areas with those of authentic standards. Furthermore, they were used as standards for semi-quantification of different phenolic categories (i.e., hydroxybenzoic acids, tyrosols, caffeoylquinic acids, flavanols, flavanol oligomers, hydroxycinnamic acids, stilbenes, anthraquinones, and flavanones), in respective.

### 3.5. Statistical Analyses

Each experiment was carried out in triplicate and data were expressed as mean ± standard deviation. The statistical significance (*t*-test: two-sample equal variance, using two-tailed distribution) was determined using SPSS software (Version 22.0, SPSS Inc., Chicago, IL, USA). *p* < 0.05 was set to be significant.

## 4. Conclusions

The present study reported for the first time a comprehensive method for screening the adulterants and alternatives for *S. cuneata*, which facilitates its industrial manufacture and should inspire other researchers in the process of screening herbal medicines with antioxidant capacities. Significantly, an efficient HPLC fingerprint was established for screening *S. cuneata*, and tyrosols and caffeoylquinic acids were considered as effective markers. *P. cuspidatum* and *B. championii* possessed similar phenolic contents and antioxidant activities but the diverse phenolic composition, indicating that they may be considered as potential candidate alternatives for *S. cuneata*. Furthermore, contents of different phenolic categories were semi-quantified and their antioxidant contributions were subsequently determined to clarify the differences between *S. cuneata* and its potential candidate alternatives, *P. cuspidatum* and *B. championii*. Nevertheless, to realize the substitution of *P. cuspidatum* and *B. championii*, cell and animal models should be further applied to validate the diversions of medical effects among *S. cuneata*, *P. cuspidatum* and *B. championii*.

## Figures and Tables

**Table 1:** Phenolic compounds tentatively identified with UPLC-DAD-QTOF-MS/MS

Peak No.	λ_max_	[M−H]^−^ (*m*/*z*)	Error (ppm)	Formula	MS/MS Fragments (*m*/*z*)	Identified Compounds	Compound Group	Samples	References
	(nm)								
1	230, 250, 294	153	−3.9	C_7_H_5_O_4_	109	Protocatechuic acid	Hydroxybenzoic acids	SC	[29]
2	255, 286	167	−2.4	C_8_H_7_O_4_	108, 123, 152		Hydroxybenzoic acids	SC	[27]
3	262	197	−9.6	C_9_H_9_O_5_	182, 166, 153	Syringic acid	Hydroxybenzoic acids	SC	[30]
4	228, 272	169	3.5	C_7_H_5_O_5_	125	Gallic acid	Hydroxybenzoic acids	PC	[31] *
5	230, 280	315	−1.6	C_14_H_19_O_8_	153, 135	Hydroxytyrosol-1-*O*-glucoside	Tyrosols	SC	[32]
6	228, 276	299	2	C_14_H_19_O_7_	137, 119, 101, 96	Salidroside	Tyrosols	SC	[43] *
7	242, 326	353	1.1	C_16_H_17_O_9_	191	3-*O*-Caffeoylquinic acid	Caffeoylquinic acids	SC	[35]
8	247, 316	353	−0.8	C_16_H_17_O_9_	191, 78, 96	5-*O*-Caffeoylquinic acid	Caffeoylquinic acids	SC	[35] *
9	235, 280	289	−0.3	C_15_H_13_O_6_	137, 109	Catechin	Flavanols	PC BC	[37]
10	234, 278	289	−0.7	C_15_H_13_O_6_	137, 109	Epicatechin	Flavanols	SC PC BC	[37] *
11	239, 280	441	−1.8	C_22_H_17_O_10_	289, 169	Catechin-gallate	Flavanols	PC	[38]
12	236, 278	577	1.2	C_30_H_25_O_12_	407, 289, 425	B type proanthocyanidin dimer 1	Flavanol oligomer	SC BC	[37] *
13	237, 279	577	5.5	C_30_H_25_O_12_	289, 407	B type proanthocyanidin dimer 2	Flavanol oligomers	BC	[43]
14	243, 279	577	−5	C_30_H_25_O_12_	287, 289	B type proanthocyanidin dimer 3	Flavanol oligomers	BC	[43] *
15	239, 278	865	2.1	C_45_H_37_O_18_	577, 695, 407, 287	B type proanthocyanidin trimer	Flavanol oligomers	SC BC	[38]
16	238, 276	579	−2.8	C_28_H_35_O_13_	417, 181, 96	Syringaresinol-4-*O*-glucoside	Lignans	SC	[22]
17	242, 280	581	10.3	C_28_H_37_O_13_	419	Lyoniresinol glucoside	Lignans	BC	[43]
18	245, 283, 330	359	−4.2	C_18_H_15_O_8_	359, 197, 161, 135	Rosmarinate	Hydroxycinnamic acids	SC	[29] *
19	245, 283	493	−4.1	C_26_H_21_O_10_	295	Salvianolic acid A	Hydroxycinnamic acids	SC	[29] *
20	244, 285	717	−1.8	C_36_H_29_O_16_	519, 321	Salvianolic acid B	Hydroxycinnamic acids	SC	[29] *
21	249, 300	469	8.3	C_20_H_21_O_11_S	227, 215	Resveratrol-sulfoglucoside	Stilbenes	PC	[43]
22	249, 304	389	3.1	C_20_H_21_O_8_	227	Resveratrol-4′-glucoside (Resveratroloside)	Stilbenes	PC	[38]
23	245, 280	469	10	C_20_H_21_O_11_S	227, 407, 289	Resveratrol-sulfoglucoside (isomer)	Stilbenes	PC	[43]
24	236, 319	389	6.4	C_20_H_21_O_8_	227	Resveratrol-3-*O*-glucoside (Piceid)	Stilbenes	PC	[38]
25	246, 300	541	3	C_27_H_25_O_12_	313, 169, 227	Resveratrol-galloylglucoside	Stilbenes	PC	[38]
26	237, 306	227	−3.5	C_7_H_15_O_8_	185, 143	Resveratrol	Stilbenes	PC	[41] *
27	254, 282, 426	431	−2.6	C_21_H_19_O_10_	269	Emodin-8-*O*-glucoside	Anthraquinones	PC	[38] *
28	229, 281, 427	431	2.3	C_21_H_19_O_10_	269	Emodin-1-*O*-glucoside	Anthraquinones	PC	[38] *
29	248, 280, 427	517	−2.7	C_24_H_21_O_13_	269, 473	Emodin-8-*O*-(6′-*O*-malonyl)-glucoside	Anthraquinones	PC	[38]
30	247, 272, 421	283	4.2	C_16_H_11_O_5_	268, 240	Physcion	Anthraquinones	PC	[38]
31	250, 268, 441	285	4.6	C_15_H_9_O_6_	267, 257, 255	Citreorosein	Anthraquinones	PC	[43]
32	249, 273, 441	299	0.7	C_15_H_7_O_7_	255, 227	Emodic acid	Anthraquinones	PC	[43]
33	267, 288, 441	269	3	C_19_H_9_O_2_	225, 241	Emodin	Anthraquinones	PC	[38] *
34	242, 279	417	10.1	C_21_H_21_O_9_	255	Liquiritin	Flavanones	BC	[37]
35	242, 279	417	4.1	C_21_H_21_O_9_	255	Neoliquiritin	Flavanones	BC	[37] *
36	244, 279	417	−1.9	C_21_H_21_O_9_	255	Neoisoliquiritin	Flavanones	BC	[35]
37	244, 279	255	5.9	C_15_H_11_O_4_	119	Liquiritigenin	Flavanones	BC	[37] *
38	247, 279	255	4.7	C_15_H_11_O_4_	119	Isoliquiritigenin	Flavanones	BC	[37] *

* Identification was verified via standards.

**Table 2:** Antioxidant capacities of nine selected phenolic compounds assessed with DPPH, ABTS, and ORAC assays.

	Categories	Antioxidant Capacities *			Semi-Contents ^#^	Contributions (%) to		
		DPPH	ABTS	ORAC		DPPH	ABTS	ORAC
*in SC*								
gallic acid	Hydroxybenzoic acids	3.98 ± 0.38 ^a^	6.08 ± 0.36 ^a^	2.23 ± 0.03 ^f^	9.19 ± 0.12	23.81 ± 3.49	31.88 ± 0.14	8.29 ± 0.01
tyrosol	Tyrosols	0.06 ± 0.02 ^f^	0.21 ± 0.04 ^e^	1.96 ± 0.03 ^g^	116.44 ± 0.44	5.60 ± 2.33	17.18 ± 0.00	113.63 ± 0.01
chlorogenic acid	Caffeoylquinic acids	0.69 ± 0.02 ^c^	0.41 ± 0.05 ^c^	5.01 ± 0.17 ^b^	112.04 ± 0.87	24.16 ± 2.24	12.58 ± 0.00	108.95 ± 0.01
epicatechin	Flavanols	1.37 ± 0.11 ^b^	0.41 ± 0.03 ^c^	4.45 ± 0.02 ^c^	21.47 ± 0.21	11.22 ± 2.36	2.94 ± 0.00	22.64 ± 0.00
proanthocyanidin B2	Flavanol oligomers	0.10 ± 0.00 ^e^	0.16 ± 0.01 ^f^	6.93 ± 0.05 ^a^	22.24 ± 0.41	0.43 ± 0.00	0.60 ± 0.00	18.32 ± 0.00
rosmarinic acid	Hydroxycinnamic acids	0.65 ± 0.10 ^c^	0.53 ± 0.05 ^b^	4.80 ± 0.06 ^b^	26.32 ± 0.26	5.26 ± 2.63	3.76 ± 0.00	24.12 ± 0.00
Sum of contributions						70.48 ± 2.08	68.95 ± 0.02	295.94 ± 0.01
*in PC*								
gallic acid	Hydroxybenzoic acids	3.98 ± 0.38 ^a^	6.08 ± 0.36 ^a^	2.23 ± 0.03 ^f^	6.8 ± 0.14	18.64 ± 2.58	29.91 ± 0.14	4.00 ± 0.01
epicatechin	Flavanols	1.37 ± 0.11 ^b^	0.41 ± 0.03 ^c^	4.45 ± 0.02 ^c^	62.62 ± 0.35	34.65 ± 6.89	10.89 ± 0.00	43.16 ± 0.00
resveratrol	Stilbenes	0.44 ± 0.01 ^d^	0.28 ± 0.11 ^d^	2.43 ± 0.03 ^e^	152.66 ± 1.18	34.51 ± 1.53	23.07 ± 0.00	73.07 ± 0.00
emodin	Anthraquinones	ND ^h^	0.22 ± 0.04 ^e^	2.50 ± 0.10 ^e^	185.11 ± 0.62	0.00 ± 0.00	1.69 ± 0.00	76.98 ± 0.00
Sum of contributions						87.80 ± 2.75	65.56 ± 0.04	197.22 ± 0.01
*in BC*								
epicatechin	Flavanols	1.37 ± 0.11 ^b^	0.41 ± 0.03 ^c^	4.45 ± 0.02 ^c^	112.39 ± 0.76	59.97 ± 12.36	15.49 ± 0.00	164.40 ± 0.00
proanthocyanidin B2	Flavanol oligomers	0.10 ± 0.00 ^e^	0.16 ± 0.01 ^f^	6.93 ± 0.05 ^a^	212.98 ± 1.52	4.16 ± 0.00	5.75 ± 0.00	243.42 ± 0.00
liquiritigenin	Flavanones	0.02 ± 0.00 ^g^	0.01 ± 0.00 ^g^	2.89 ± 0.05 ^d^	21.01 ± 0.08	0.19 ± 0.00	0.08 ± 0.00	22.61 ± 0.00
Sum of contributions						64.32 ± 4.12	21.32 ± 0.00	430.42 ± 0.00

* indicated that results were presented as μmol Trolox equivalent/μmol phenolic compound, different superscript letters in each row means significant difference. # presented the results as mg phenolic compound/g d.w., respectively.

**Table 3:** Collected herbs: *S. cuneata* and its six adulterants.

Abbr.	Species	Collection Site	Collection Date	Material Conditions
SC	*Sargentodoxa cuneata* (Oliv.) Rehd. et Wils.	Beijing	2015.7.26	dry slices
PC	*Polygonum cuspidatum* Sieb.et Zucc.	Anguo, Heibei	2015.8.30	dry slices
BC	*Bauhinia championii* (Benth.) Benth.	Anguo, Heibei	2015.8.30	dry slices
SS	*Schisandra sphenanthera* Rehd. et Wils.	Anguo, Heibei	2015.8.30	dry slices
SG	*Schisandra grandiflora* (Wall.) Hook. f. et Thoms	Anguo, Heibei	2015.8.30	dry slices
MD	*Millettia dielsiana* Harms.	Anguo, Heibei	2015.8.30	dry slices
RC	*Rubia cordifolia* L.	Anguo, Heibei	2015.8.30	dry slices

## Supplementary Materials

Supplementary materials can be found at https://www.mdpi.com/1422-0067/20/21/5427/s1.

## Abbreviations

DPPH	2,2′-diphenyl-1-picrylhydrazyl
ABTS	2,2′-azobis-(3-ethylbenzothiazoline-6-sulfonic acid)
ORAC	oxygen radical absorbance capacity
AAPH	2,2′-azobis (2-methylpropionamidine) dihydrochloride
UPLC-QTOF-MS/MS	ultra performance liquid chromatography-diode array detector-quadrupole time-of-flight-mass spectrometer/mass spectrometer
HPLC	high performance liquid chromatography
GAE	gallic acid equivalent
RTE	rutin equivalent
TE	Trolox equivalent
ROS	reactive oxygen species
SC	*Sargentodoxa cuneata*
PC	*Polygonum cuspidatum*
BC	*Bauhinia championii*
SS	*Schisandra sphenanthera*
SG	*Schisandra grandiflora*
MD	*Millettia dielsiana*
RC	*Rubia cordifolia*
