# Supplementary material for: Screen for Potential Candidate Alternatives of Sargentodoxa cuneata from Its Six Adulterants Based on Their Phenolic Compositions and Antioxidant Activities
Source: Int J Mol Sci. 2019 Oct 31;20(21):5427. doi: 10.3390/ijms20215427 (PMC6862427; doi:10.3390/ijms20215427)
Supplement: Supplementary file 1 [file ijms-20-05427-s001.pdf]

Supplementary Materials

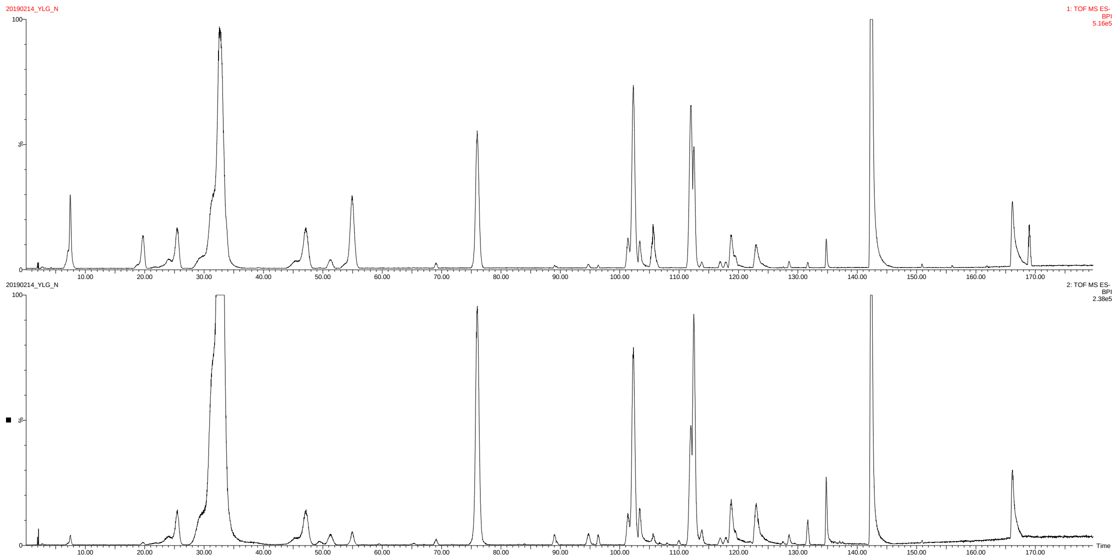

Supplementary Figure 1a. Total ion chromatograph of MS and MS/MS data of mixed standards.

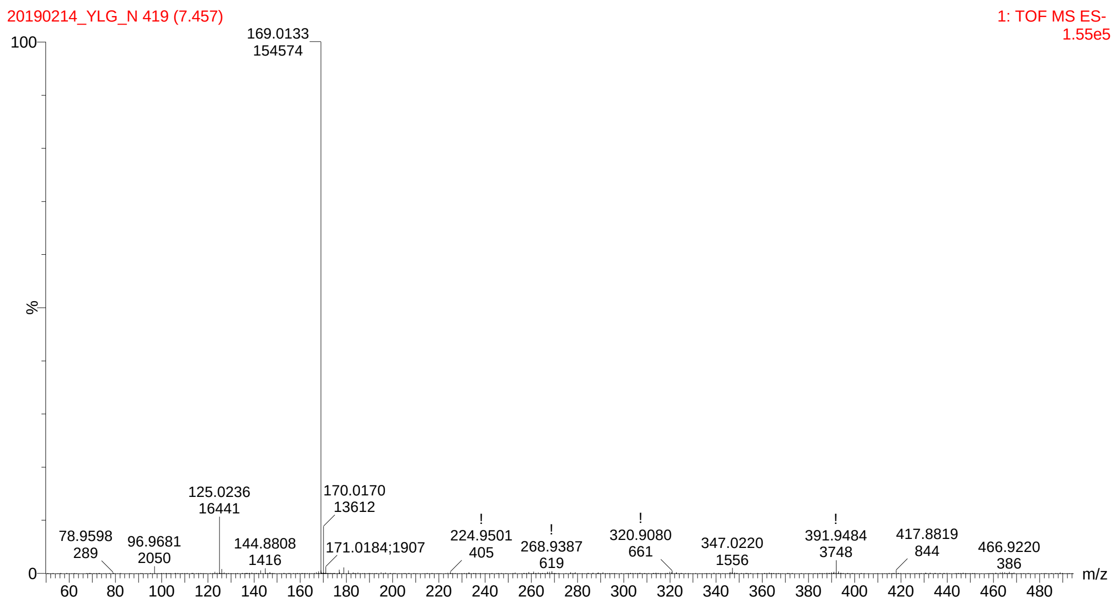

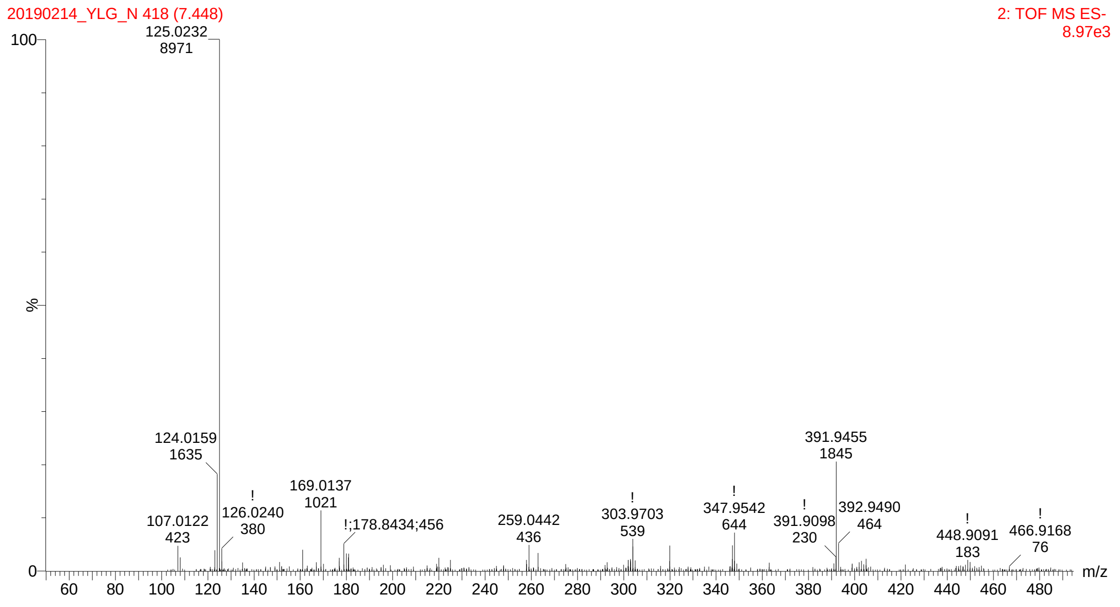

Supplementary Figure 1.b. MS and MS/MS fragments of compound 4, i.e., gallic acid.

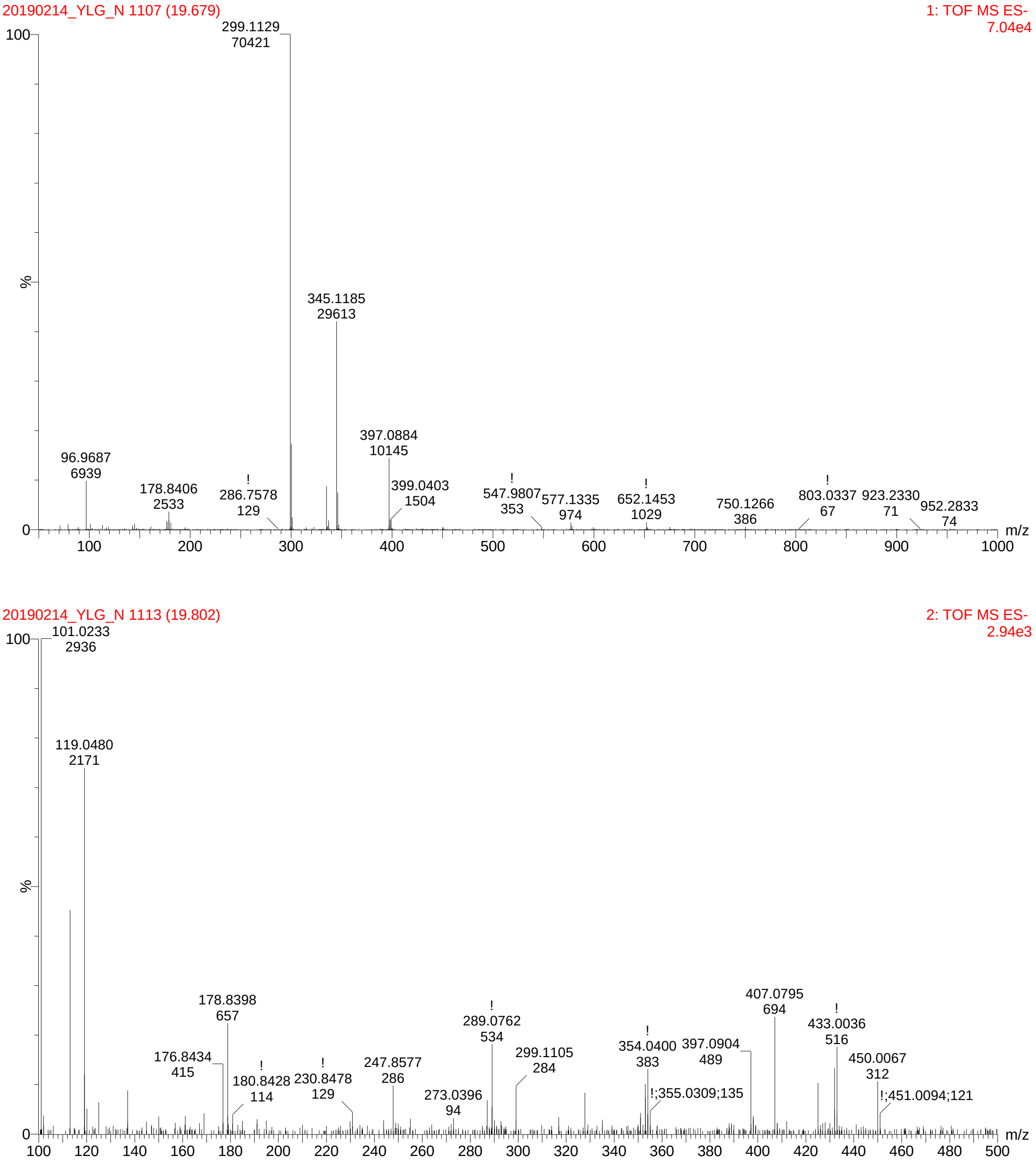

Supplementary Figure 1c. MS and MS/MS fragments of compound 6, i.e., salidroside.

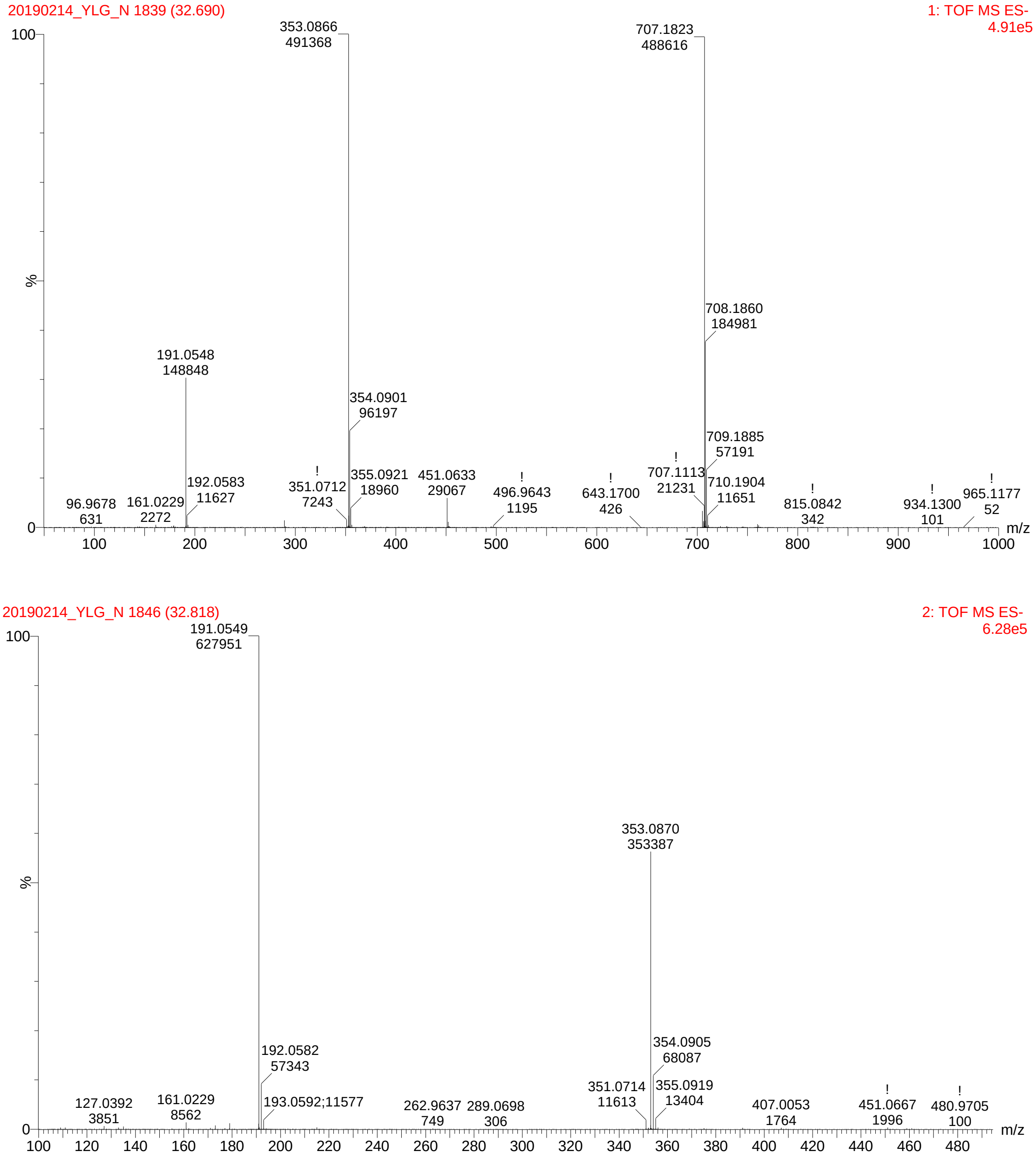

Supplementary Figure 1d. MS and MS/MS fragments of compound 8, i.e., 5-*O*-caffeoylquinic acid.

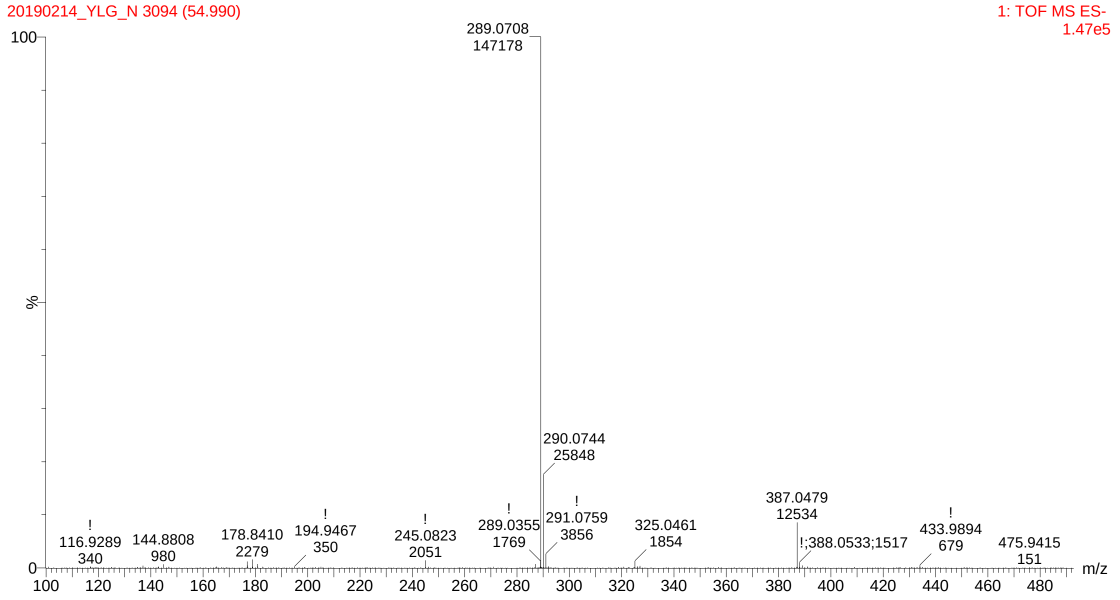

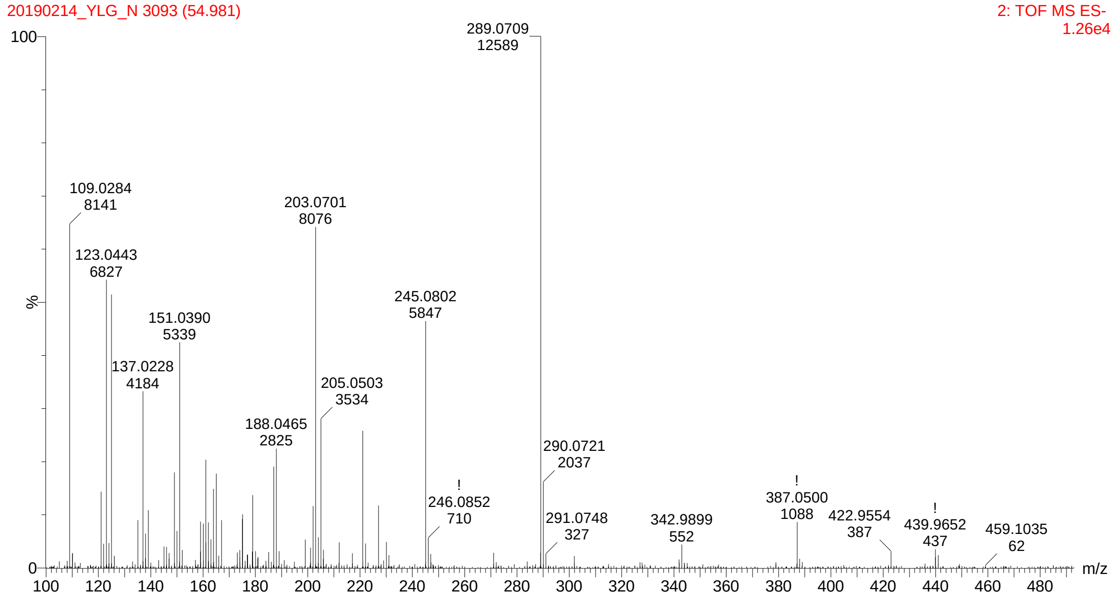

Supplementary Figure 1e. MS and MS/MS fragments of compound 10, i.e., epicatechin.

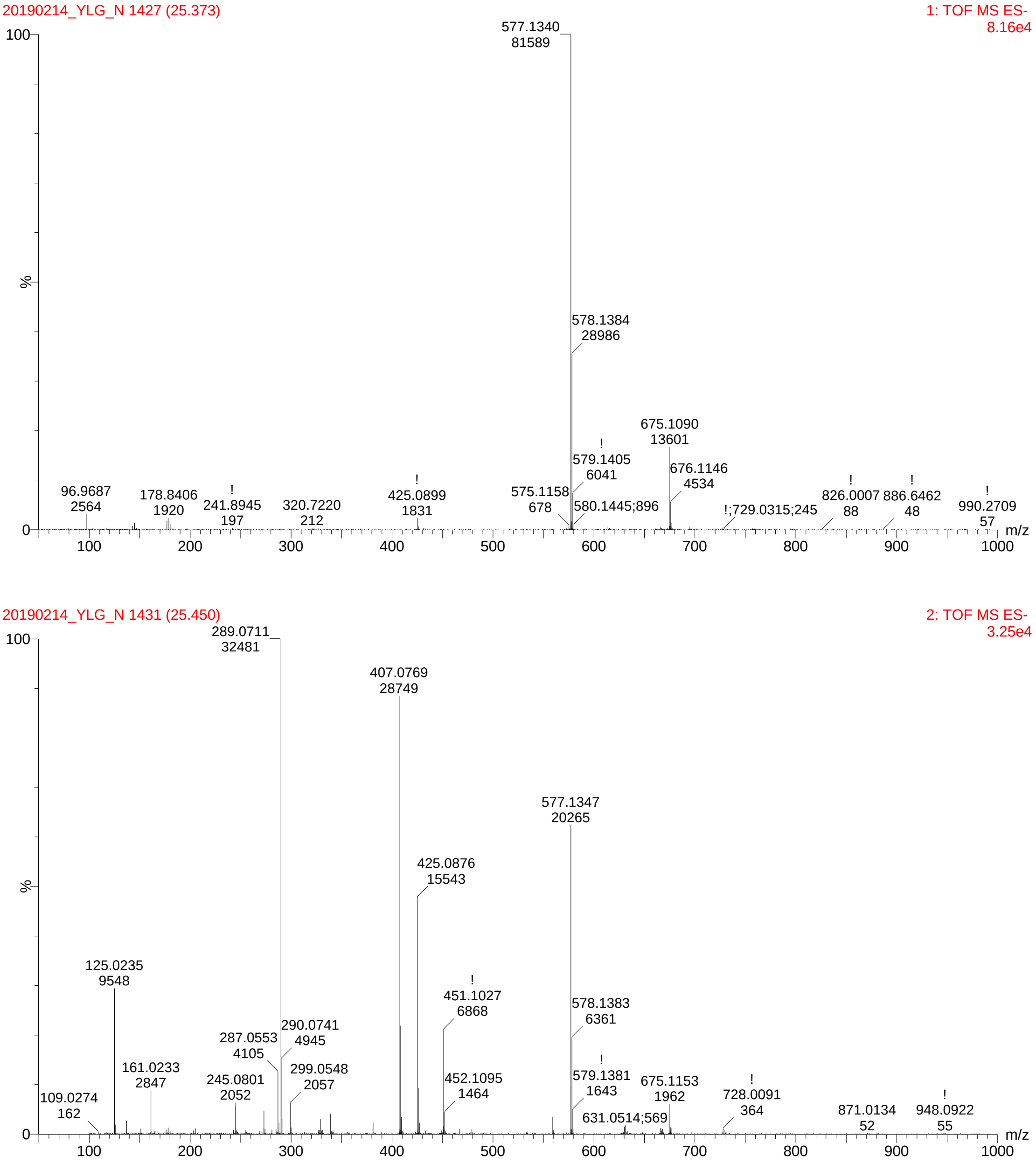

Supplementary Figure 1f. MS and MS/MS fragments of compound 12, i.e., B type proanthocyanidin dimer 1.

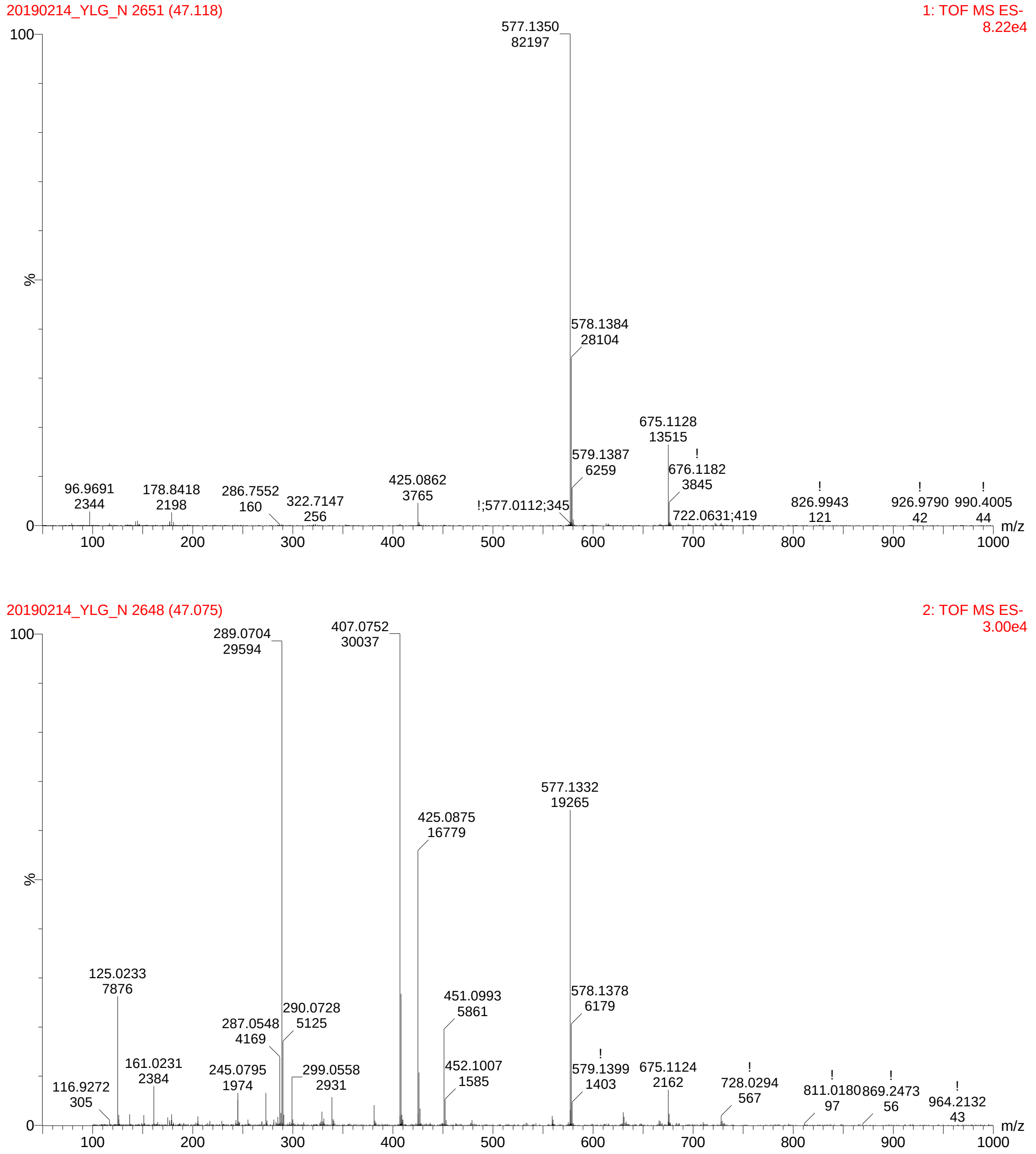

Supplementary Figure 1g. MS and MS/MS fragments of compound 14, i.e., B type proanthocyanidin dimer 3.

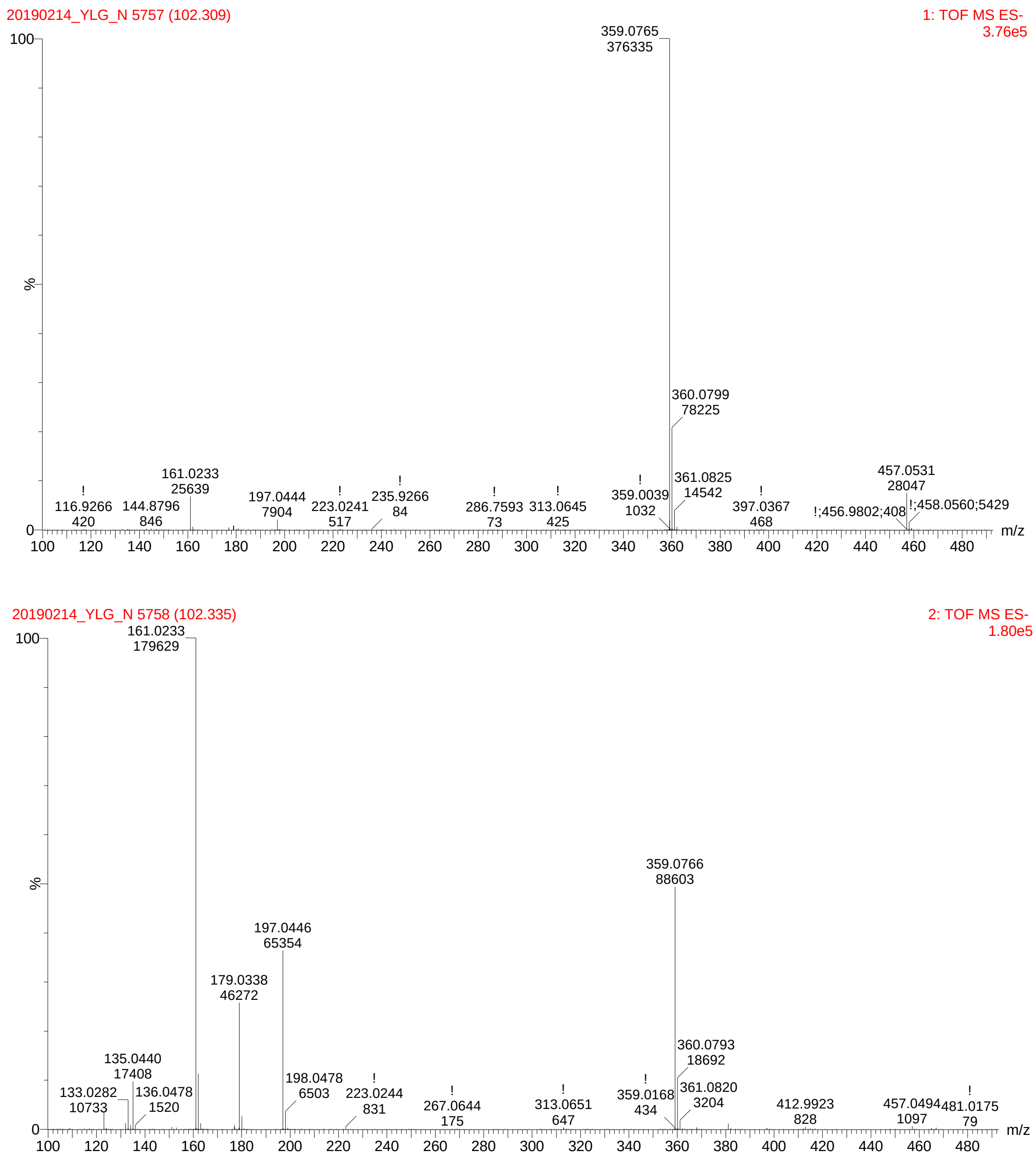

Supplementary Figure 1h. MS and MS/MS fragments of compound 18, i.e., rosmarinate.

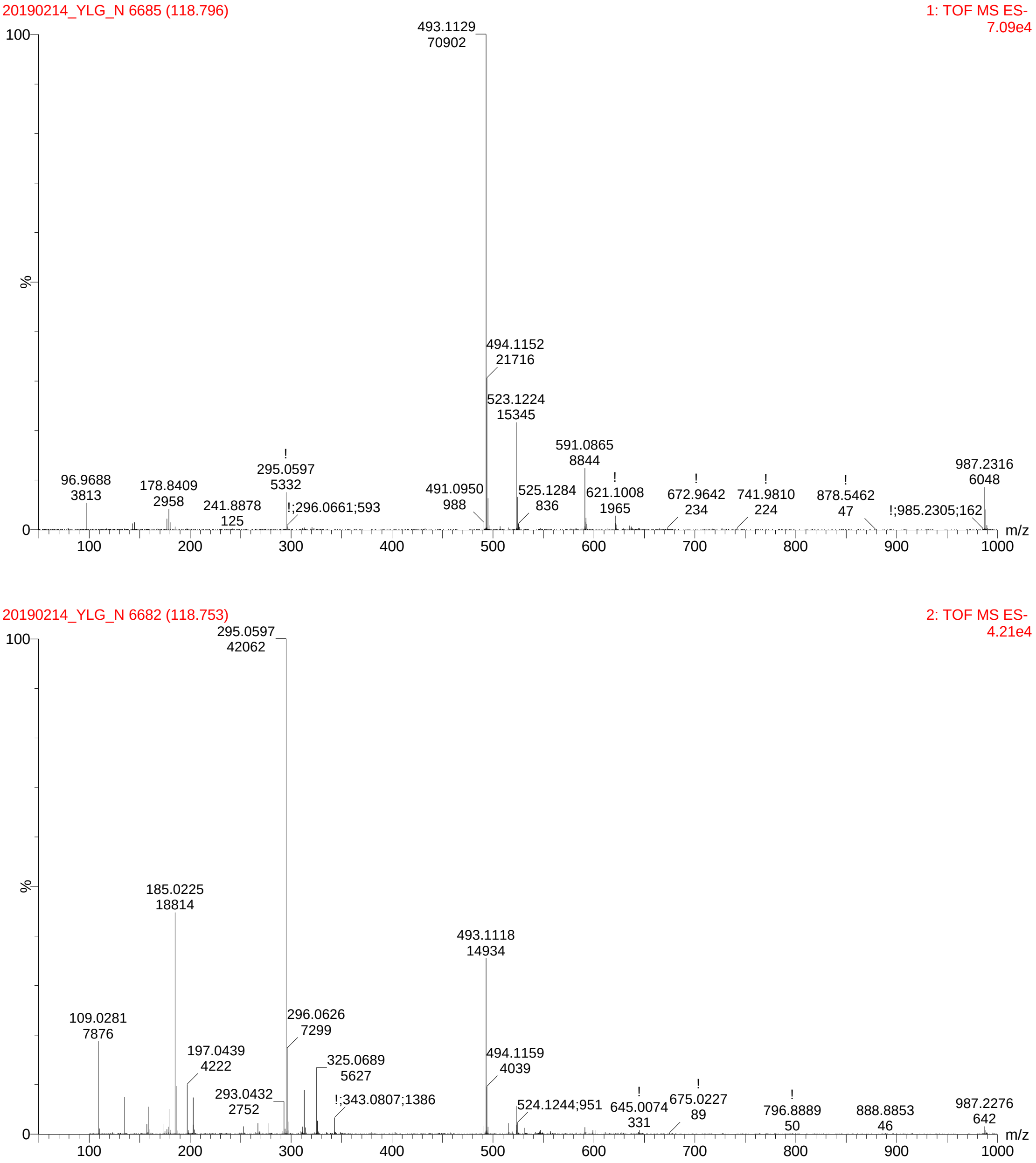

Supplementary Figure 1i. MS and MS/MS fragments of compound 19, i.e., salvianolic acid A.

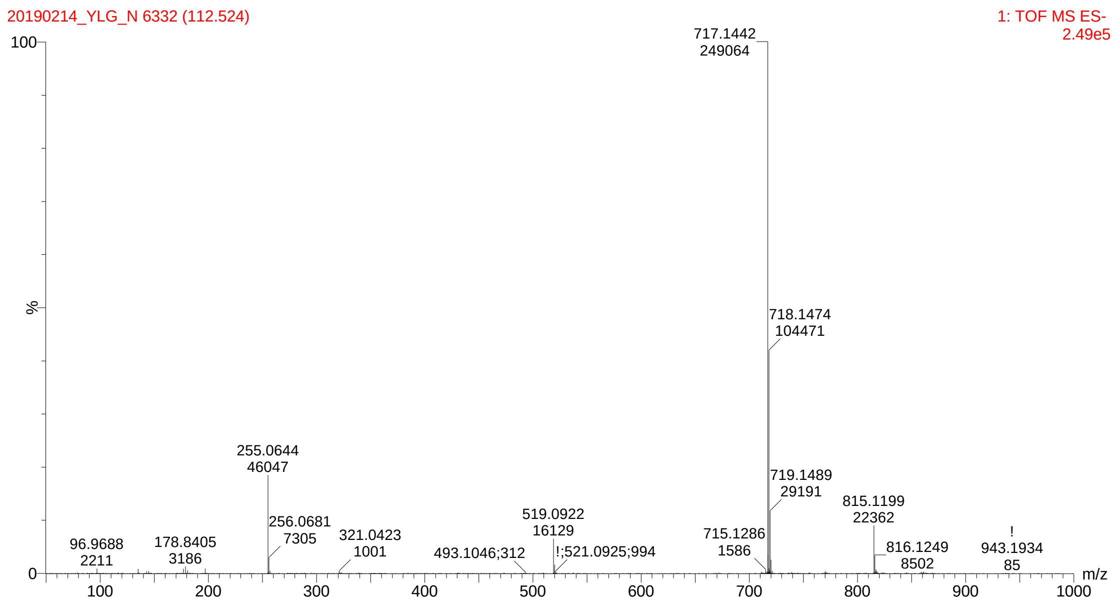

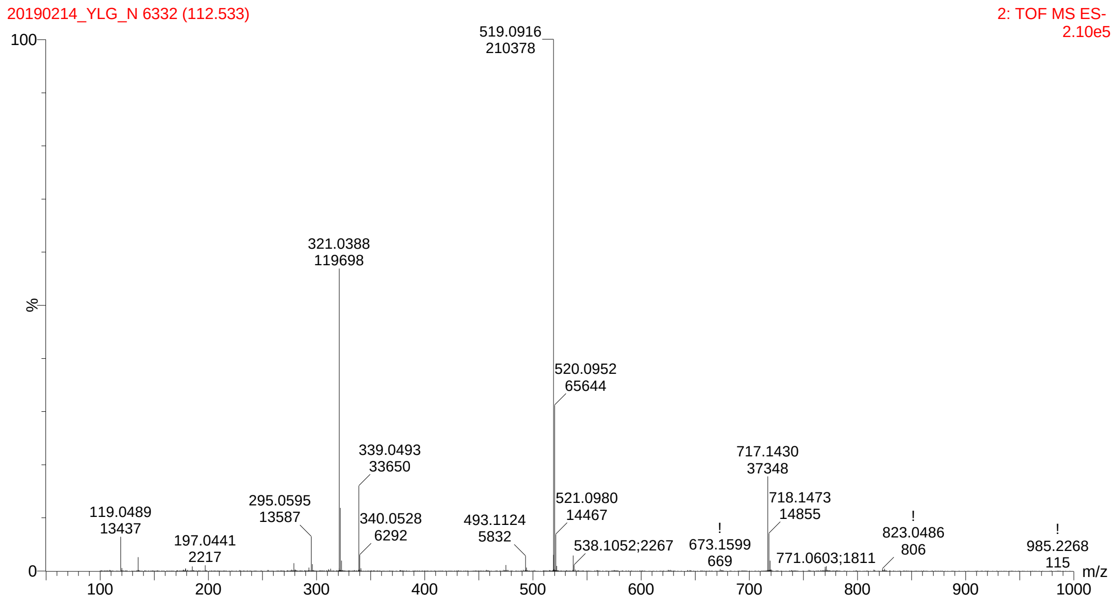

Supplementary Figure 1j. MS and MS/MS fragments of compound 20, i.e., salvianolic acid B.

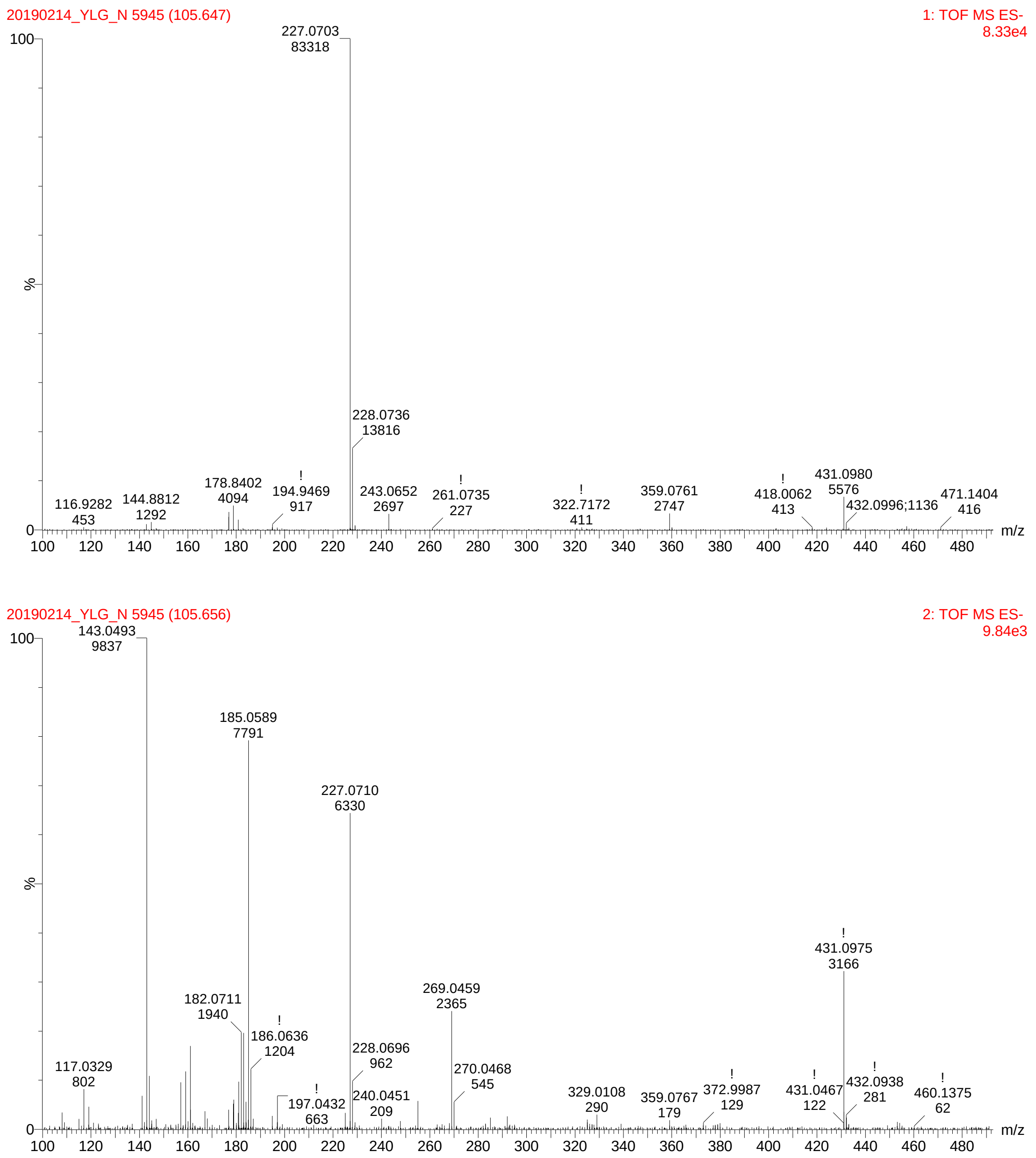

Supplementary Figure 1k. MS and MS/MS fragments of compound 26, i.e., resveratrol.

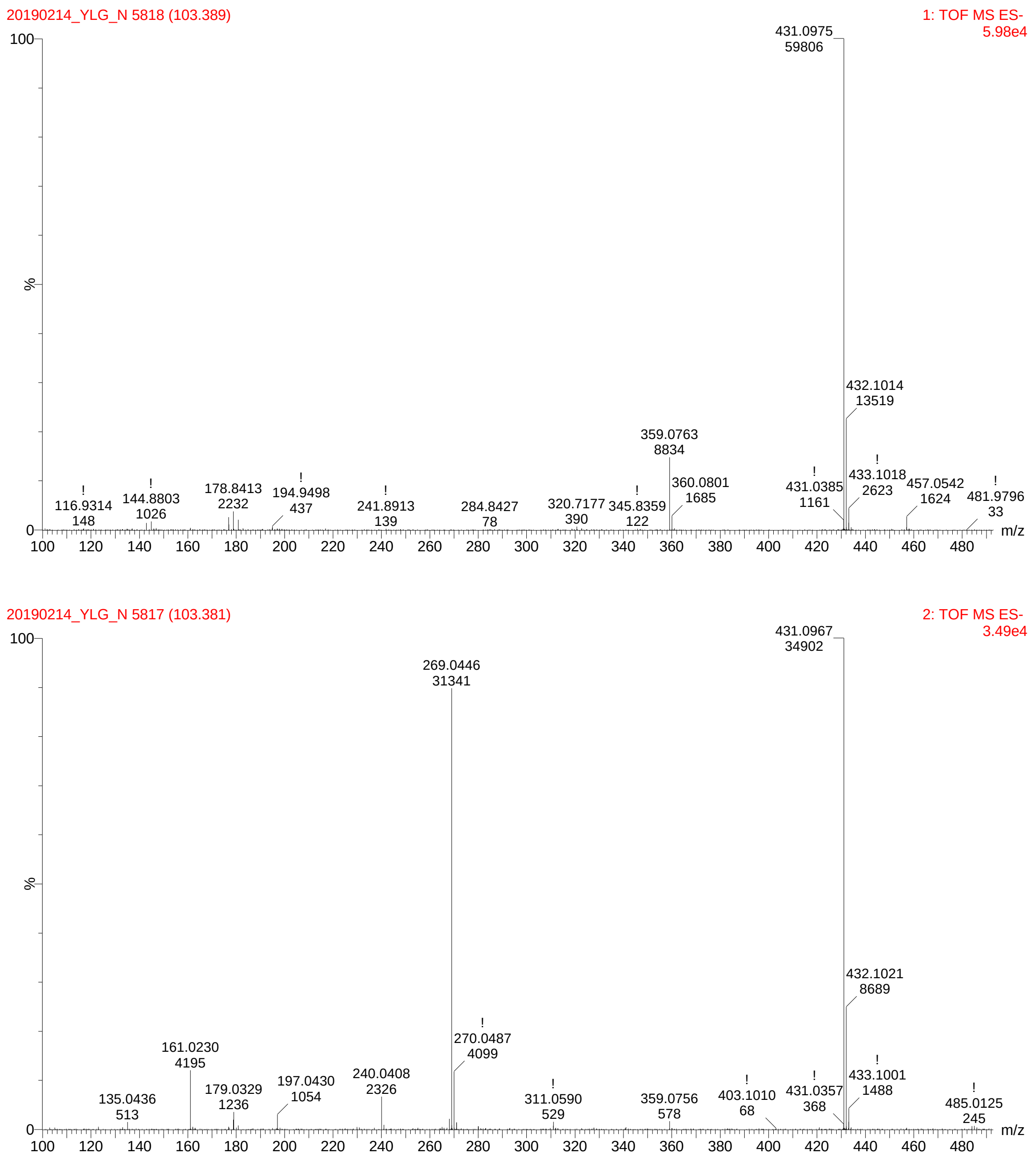

Supplementary Figure 1L. MS and MS/MS fragments of compound 27, i.e., emodin-8-*O*-glucoside.

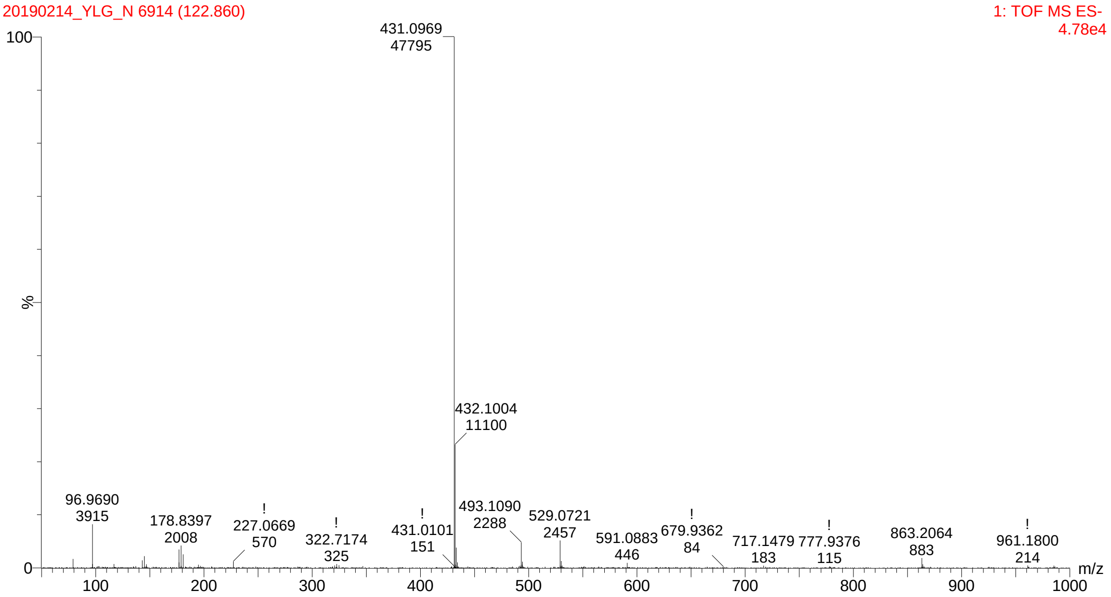

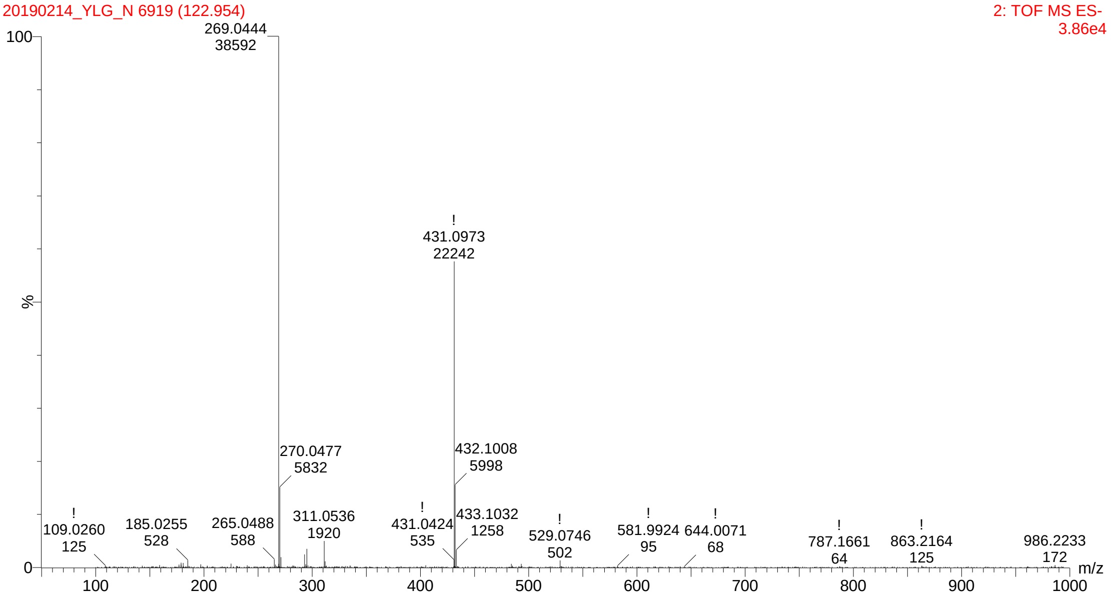

Supplementary Figure 1m. MS and MS/MS fragments of compound 28, i.e., emodin-1-*O*-glucoside.

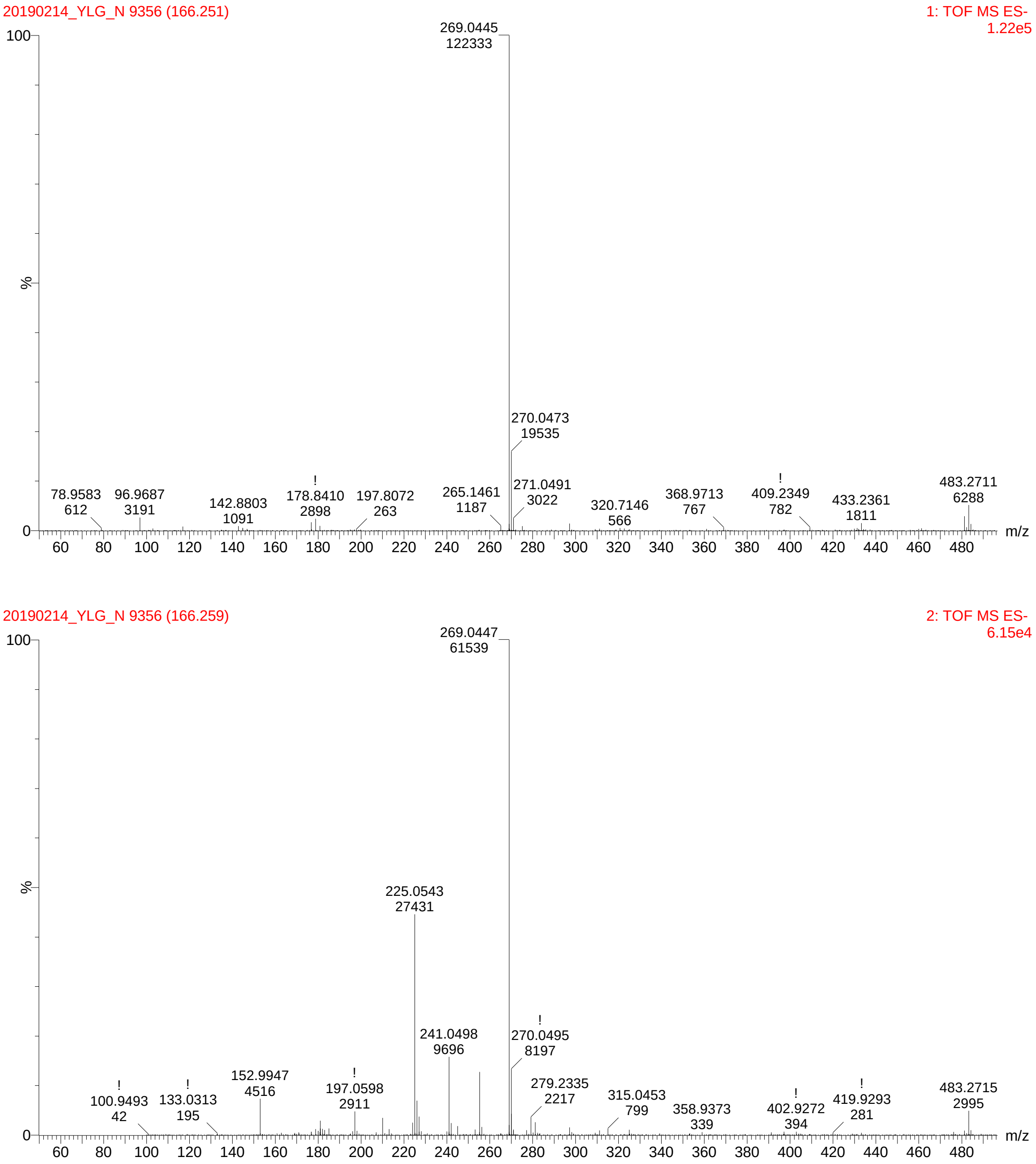

Supplementary Figure 1n. MS and MS/MS fragments of compound 33, i.e., emodin.

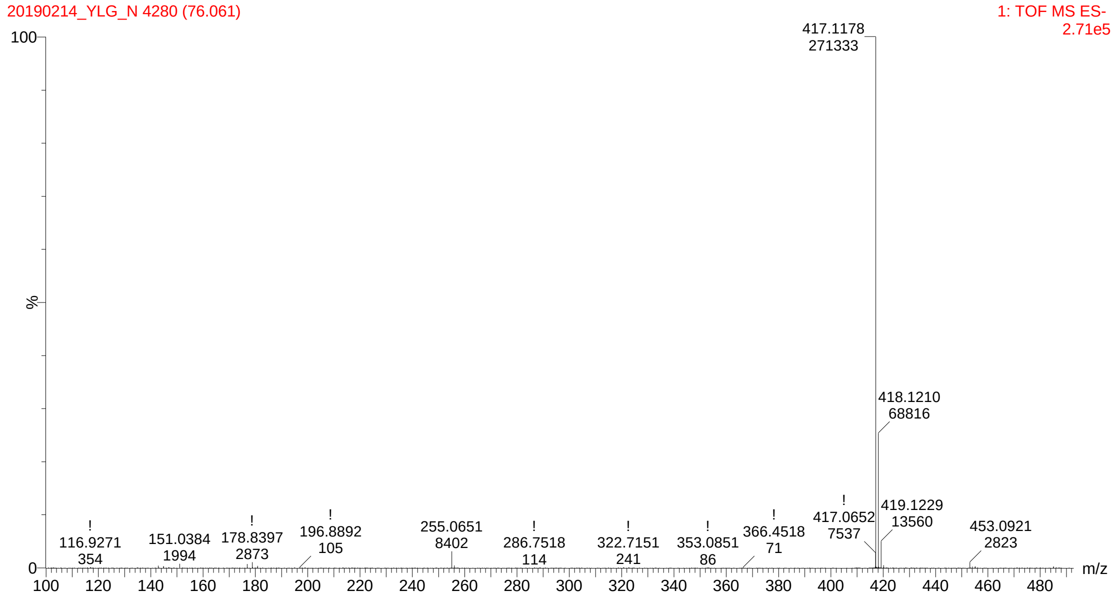

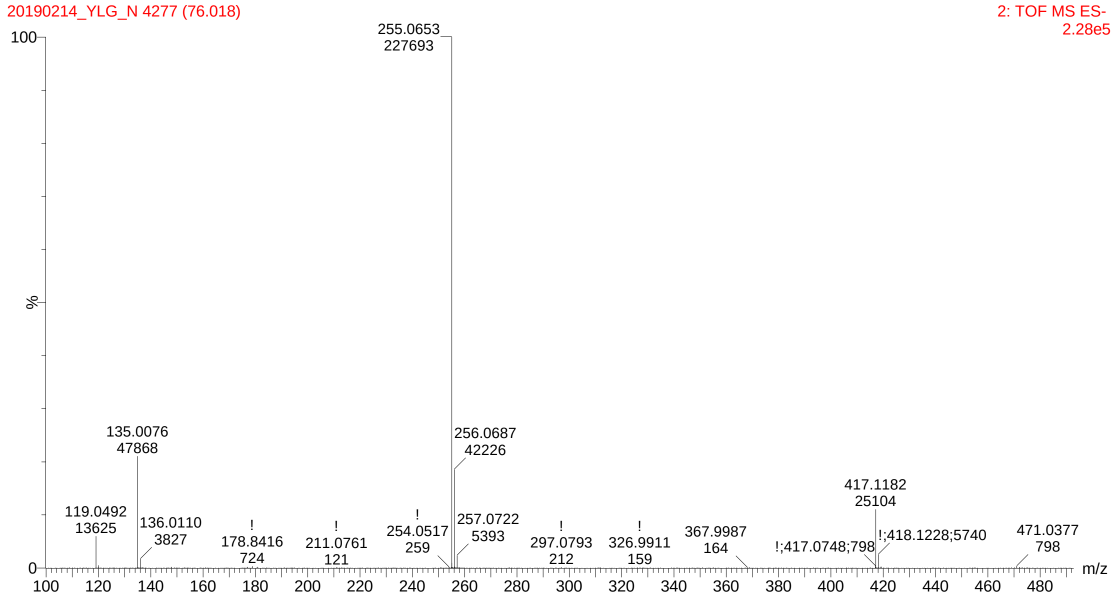

Supplementary Figure 1o. MS and MS/MS fragments of compound 35, i.e., neoliquiritin.

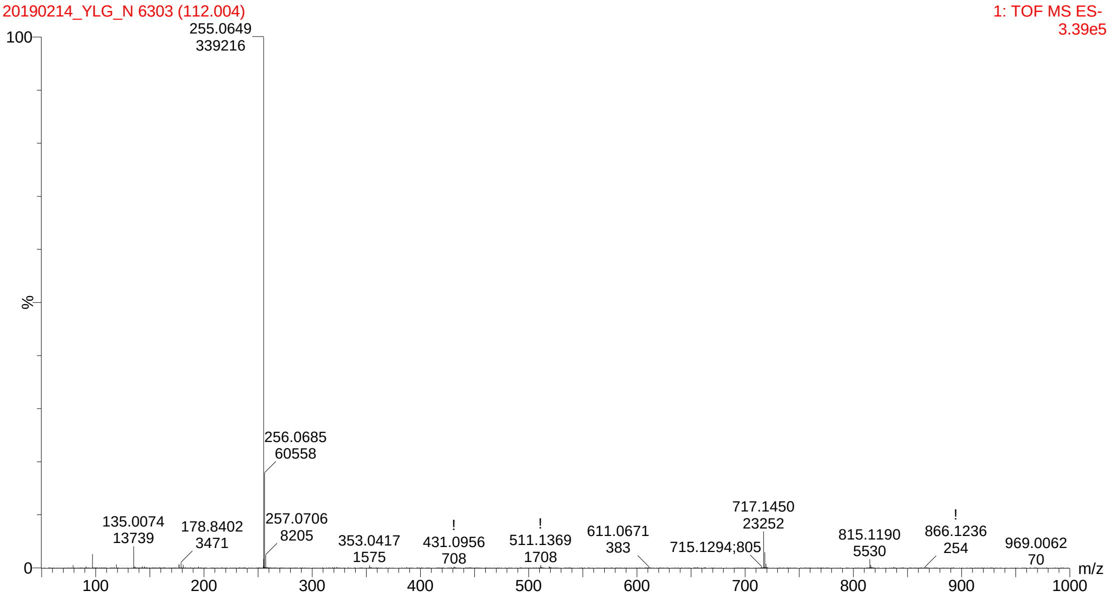

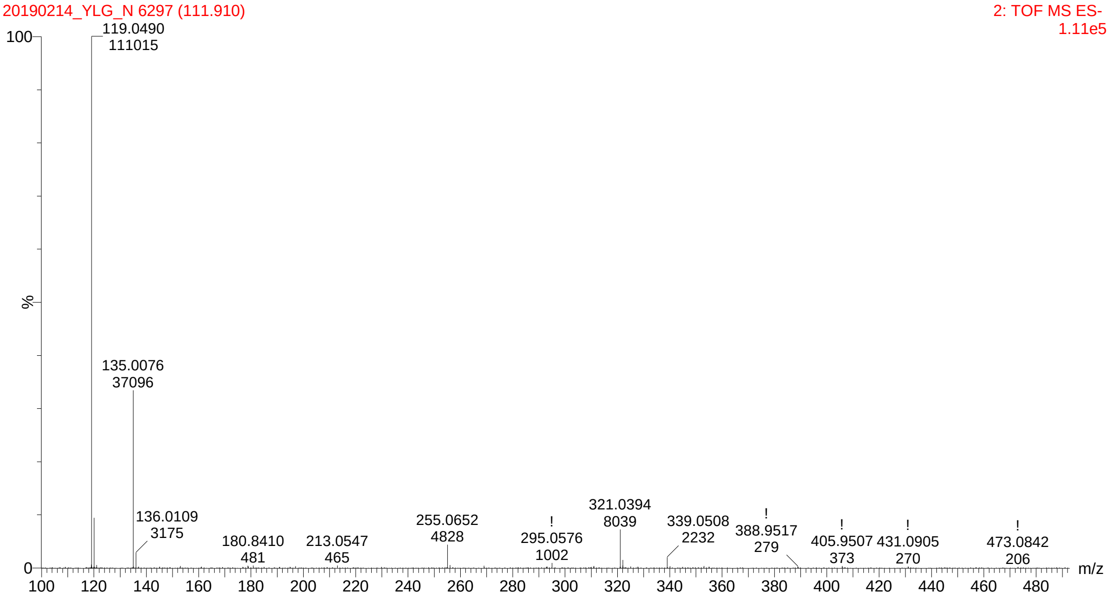

Supplementary Figure 1p. MS and MS/MS fragments of compound 37, i.e., liquiritigenin.

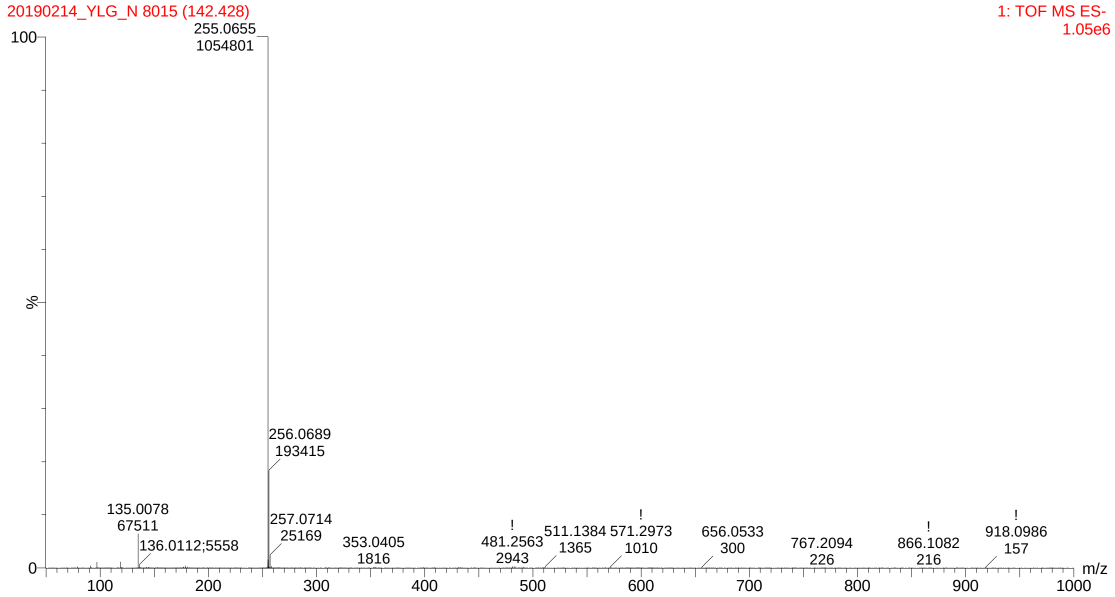

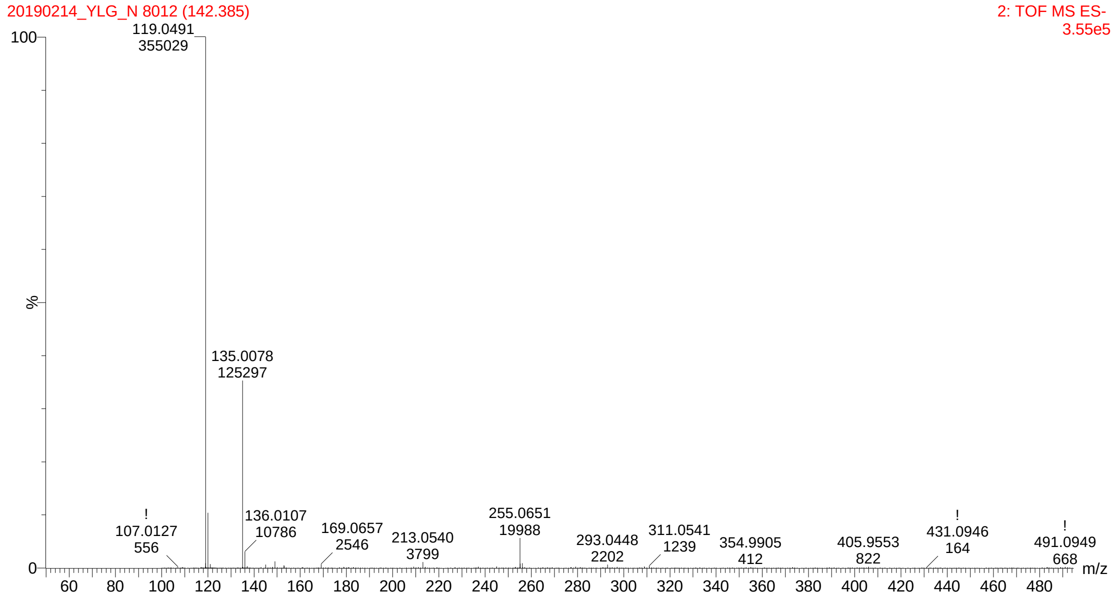

Supplementary Figure 1q. MS and MS/MS fragments of compound 38, i.e., isoliquiritigenin.
